# Single-cell spatial transcriptomics reveals hepatocyte reprogramming in Fontan-associated liver disease

**DOI:** 10.1172/jci.insight.198823

**Published:** 2026-02-24

**Authors:** Brandon M. Lehrich, Jordann N. Lewis, Vik Meadows, Lori Schmitt, Mylarappa B. Ningappa, Jia-Jun Liu, Silvia Liu, Catherine K. Gestrich, Victor O. Morell, Rakesh Sindhi, Satdarshan P. Monga, Anita Saraf

**Affiliations:** 1 Organ Pathobiology and Therapeutics Institute, and; 2 Department of Pharmacology and Chemical Biology, University of Pittsburgh School of Medicine, Pittsburgh, Pennsylvania, USA.; 3 Pittsburgh Liver Research Center, University of Pittsburgh and University of Pittsburgh Medical Center, Pittsburgh, Pennsylvania, USA.; 4 Medical Scientist Training Program, University of Pittsburgh, Pittsburgh, Pennsylvania, USA.; 5 Heart Institute, UPMC Children’s Hospital of Pittsburgh, Department of Pediatrics, Pittsburgh, Pennsylvania, USA.; 6 UPMC Heart and Vascular Institute, Department of Medicine, Pittsburgh, Pennsylvania, USA.; 7 Department of Pathology, University of Pittsburgh, Pittsburgh, Pennsylvania, USA.; 8 Division of Cardiothoracic Surgery, Department of Surgery, and; 9 Center for Computational Immunogenetics and Drug Repurposing (CCIDR), Pediatric Transplant Surgery, University of Pittsburgh, Pennsylvania, USA.; 10 Division of Gastroenterology, Hepatology and Nutrition, Department of Medicine, University of Pittsburgh School of Medicine, Pittsburgh, Pennsylvania USA.; 11 Division of Cardiology, Department of Medicine, University of Pittsburgh, Pittsburgh, Pennsylvania, USA.; 12 RK Mellon Institute for Pediatric Research, Department of Pediatrics, Pittsburgh, Pennsylvania, USA.

**Keywords:** Cardiology, Hepatology, Metabolism, Cardiovascular disease, Cell stress, Cellular senescence

## Abstract

Fontan-associated liver disease (FALD) is a frequent complication in single-ventricle patients palliated with the Fontan operation. FALD severity can impact clinical decisions; however, the pathophysiology of FALD progression is unknown. Single-cell spatial transcriptomics (ST) was performed on liver explant tissue sections from FALD patients with early (*n* = 1) and advanced fibrosis (*n* = 1) using CosMx Spatial Molecular Imaging with in situ hybridization of 6000 genes. Immunofluorescence for liver zonation and cellular stress markers was performed to confirm protein expression based on ST analysis in additional FALD tissues (*n* = 18). Unbiased clustering yielded 12 liver cell types, comprising 6 subtypes of hepatocytes. FALD with advanced fibrosis demonstrated expansion of mid-zonal hepatocytes, accompanied by loss of zonal markers characteristic of canonical pericentral and periportal hepatocytes. A subset of hepatocytes in advanced FALD demonstrated increased cellular stress and a redundant zonal phenotype, which we have termed zonally ambiguous and stressed hepatocytes. CellChat analysis revealed that ectopic WNT2 signaling is likely driving disrupted hepatocyte zonation. To corroborate these bioinformatic findings, we performed immunofluorescent staining of FALD specimens, which confirmed a disruption of liver zonation, and a significant increase in heat shock protein 70 (HSP70). Lastly, HSP70 expression strongly correlated with the congestive hepatic fibrosis (CHF) score. Thus, single-cell ST has identified a population of hepatocytes with features of cellular stress and redundant zonal gene expression specific to advanced FALD. Further studies on hepatocyte metabolic function in Fontan patients will lead to a greater understanding of FALD development and progression during chronic maladaptation.

## Abstract 2

Fontan associated liver disease (FALD) is a progressive seqalae of single ventricle Fontan physiology associated with hepatic congestion, fibrosis and cirrhosis. Spatial transcriptomics analysis of FALD tissue identified a unique population of metabolically exhausted hepatocytes unique to FALD with advanced fibrosis

## Graphical abstract

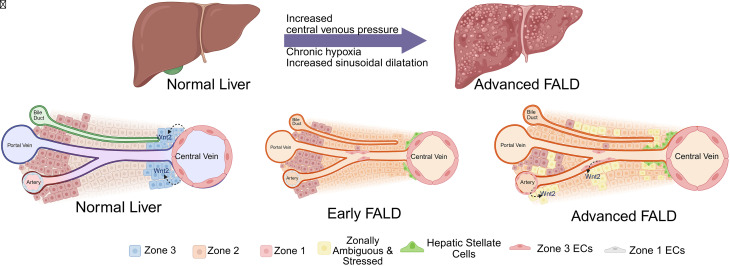

## Introduction

Single-ventricle congenital heart disease is a rare and lethal cardiac abnormality that occurs in 4–8 per 10,000 births (1). The cardiac anatomy of babies born with single-ventricle pathologies is unable to support both pulmonary and systemic circulation simultaneously, leading to cyanosis, respiratory distress, and a failure to thrive. Resultingly, without any intervention, greater than 90% of neonates with single-ventricle physiology die within the first few days of life. Over the last 50 years, an evolving series of palliative surgeries have been transformative in improving survival in these neonates. In the resulting “Fontan circulation,” pulmonary flow bypasses the subpulmonary ventricle and connects directly to the pulmonary circulation, and the pumping cardiac chamber facilitates high-pressure systemic perfusion alone. While the resulting physiology is synthetic, it has been instrumental in extending the life span of these children into many decades. For the first time, over 80% of children now survive to 18 years of life (2). There are an estimated 50,000–70,000 people globally living with a Fontan circulation, which is expected to nearly double in about 20 years (3).

However, despite the long-term survival of these children, patients with Fontan circulation develop multiple systemic comorbidities over their life span due to their unique physiology. The passive pulmonary flow resulting from the lack of pulsatile subpulmonary perfusion leads to a chronic increase in central venous pressure. Resultingly, the chronic central venous congestion transmits increased pressures into the liver, which over a lifetime leads to changes in the hepatic parenchyma in all patients with Fontan physiology. Fontan-associated liver disease (FALD) (4) increases the risk of liver fibrosis, cirrhosis, and liver lesions (i.e., hepatocellular carcinoma, hepatic adenomas, or focal nodular hyperplasia), necessitating a combined heart-liver transplantation approach in select patients (5–7).

The cellular mechanisms driving liver fibrosis in FALD are known to be distinct from those observed in patients with other forms of chronic liver injury, including metabolic dysfunction–associated steatohepatitis (MASH) and alcoholic liver disease. In FALD, the anatomical changes result in increased central venous pressures, hypoxia, sinusoidal dilation, and stiffness, which leads to a histologically distinct pattern of centrilobular fibrosis (8). Both TGF-β and HIF1α have been proposed as drivers of centrilobular fibrosis in FALD due to their upregulation following sinusoidal stretch (9, 10). However, our understanding is limited due to the rarity of FALD, and thus a lack of sufficiently powered large-scale omics studies, along with a lack of small and large animal models to validate these mechanisms.

In this study, we utilize an image-based spatial transcriptomic platform, the CosMx Spatial Molecular Imager (SMI), to generate a single-cell spatial transcriptomic atlas in human FALD. We first define cell-type heterogeneity and shifts across varying stages of fibrosis in human FALD and map these cell types spatially. Interestingly, we observed an expansion of mid-zonal and a loss of pericentral and periportal hepatocytes in advanced FALD. Thus, we postulated there was a hepatocyte subpopulation that emerged that may compensate to fulfill demand. We identified a hepatocyte subpopulation that develops in FALD, whereby hepatocytes no longer show discrete compartmentalization of gene expression across the hepatic lobule, termed “liver zonation”; instead, hepatocytes simultaneously expressed markers of multiple zones. These hepatocytes, which are stressed due to the expanded transcriptional demand, and ambiguous in expression of zonation markers, we have termed “zonally ambiguous and stressed” hepatocytes. We validated these findings using tissue biopsy and explant specimens across different stages of fibrosis in human FALD with multiplex immunofluorescence. Overall, this study proposes mechanistic findings distinguishing early and advanced fibrosis in FALD.

## Results

### A single-cell spatial transcriptomic atlas of human FALD with early and advanced fibrosis.

**Figure 1:**
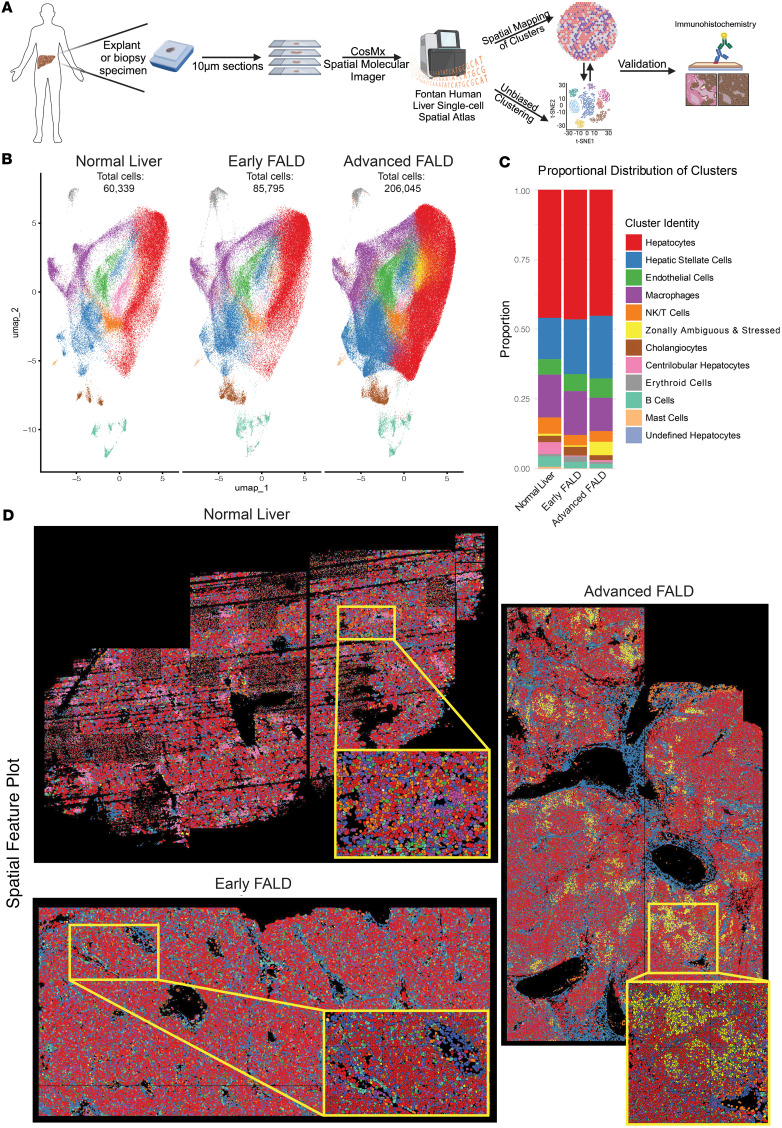
Single-cell spatial transcriptomic atlas of human FALD with early and advanced fibrosis. (**A**) Schematic diagram of workflow to develop single-cell spatial transcriptomic atlas of human FALD and data analysis pipeline. Figure created in BioRender. (**B**) Uniform manifold approximation and projection (UMAP) split by disease pathology across all annotated cell types. (**C**) Stacked bar plot indicating relative cell type proportions across the whole dataset by disease pathology colored by cell type annotation. (**D**) Spatial feature plots of all annotated cell types directly on sequenced tissue section by disease pathology.

Recent work has described intrahepatic biological differences underlying early and advanced fibrosis in FALD (11). Compared with FALD patients with early fibrosis, FALD patients with advanced fibrosis upregulate pathways associated with inflammation, angiogenesis, and hepatic congestion (11). However, the cellular composition, cell-to-cell interactions, and cellular spatial localization in FALD across varying degrees of fibrosis remains largely unknown. Here, through screening of liver explant tissues available from the FALD patients at the University of Pittsburgh Medical Center (UPMC), we aimed to develop a single-cell spatial transcriptomic atlas using the CosMx SMI platform with in situ hybridization of 6175 genes (Figure 1A). We identified 2 patients, one with early fibrosis (59 years old) and one with advanced fibrosis (21 years old) (Supplemental Figure 1, A–C; supplemental material available online with this article; https://doi.org/10.1172/jci.insight.198823DS1). The control biopsy sample was obtained, before perfusion, from a 4-year-old identified for liver donation with a congestive hepatic fibrosis (CHF) score of 0. After cell segmentation, assessment of quality control metrics, and data integration across pathologies, cells with high-quality reads (60,339 for control and 291,840 for FALD) were utilized for downstream analyses.

We utilized the Harmony data integration algorithm (12) to integrate the cellular spatial transcriptomes of the normal liver along with the early and advanced FALD livers (Supplemental Figure 2A). We then performed unbiased dimensionality reduction and clustering on all 352,179 cells, which resulted in 12 annotated cell types: hepatocytes, hepatic stellate cells (HSCs), endothelial cells, macrophages, NK/T cells, “zonally ambiguous and stressed hepatocytes,” cholangiocytes, centrilobular hepatocytes, erythroid cells, B cells, mast cells, and undefined hepatocytes (Supplemental Figure 2, B and C), based on previously reported marker genes (13). The number of genes and detected transcripts was similar across the cell clusters (Supplemental Figure 2, D and E). Each of these cell types had distinct gene expression signatures, which defined their identity following differential gene expression per cell cluster (Supplemental Figure 3A and Supplemental Figure 4A). Cell type composition differed between normal liver, early, and advanced FALD (Figure 1B and Supplemental Figure 3B). Notably, centrilobular hepatocytes, characterized by high expression of *GLUL*, *CYP2E1*, *OAT*, and *AXIN2*, decreased in number as the disease progressed, becoming nearly nonexistent in advanced FALD. Additionally, HSCs, defined by high expression of *TAGLN*, *COL6A2*, and *DCN*, expanded in advanced FALD (Figure 1C). Spatial plots corroborated these findings, where central veins were lined by HSCs in early FALD, which further expanded in advanced FALD (Figure 1D). Further analysis showed an increasing population of HSCs with myofibroblast phenotype with a relative decrease in quiescent HSCs in advanced FALD (Supplemental Figure 5A). Upregulated markers associated with these distinct cellular subtypes are illustrated in Supplemental Figure 5, B and C. Additionally, the normal liver architecture with identifiable portal triads was no longer present, with the appearance of hepatic nodules within cirrhotic areas (Figure 1D). Thus, advanced fibrosis in FALD is characterized by increases in HSCs surrounding central veins.

### A subset of hepatocytes are both zonally ambiguous and stressed and distributed across the entire lobule in advanced FALD.

**Figure 2:**
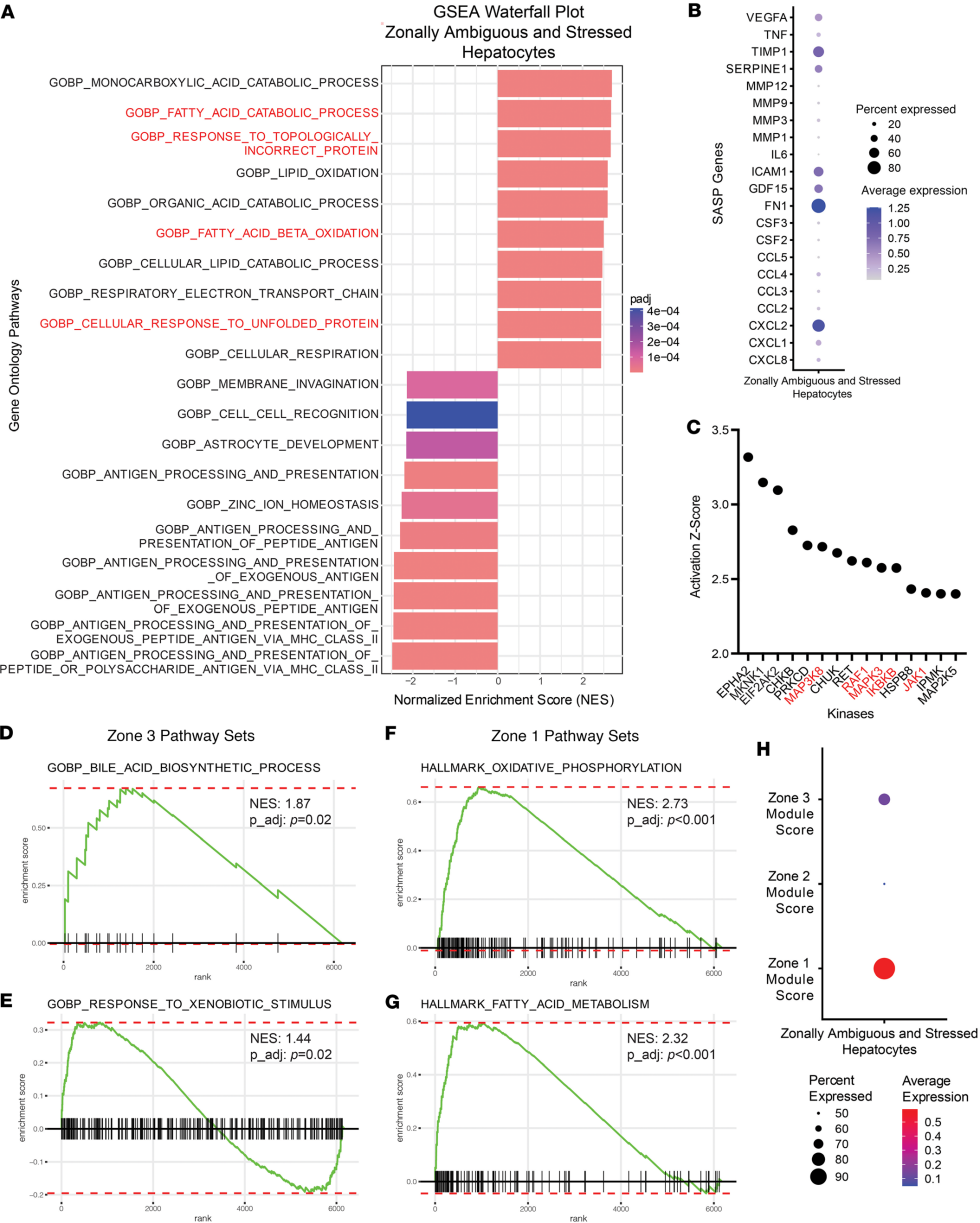
Characterization of zonally ambiguous and stressed hepatocytes demonstrates markers of cellular stress, senescence, and liver zonation. (**A**) Waterfall plot of top 10 upregulated and downregulated Gene Ontology (GO) pathways by normalized enrichment score (NES) and adjusted *P* value. (**B**) Dot plot illustrating expression of various senescence-associated secretory phenotype marker genes. (**C**) Waterfall plot of top kinases predicted to be activated based on differentially expressed genes. (**D**) Gene set enrichment analysis (GSEA) running enrichment score plot for “GO: Bile Acid Biosynthetic Process.” (**E**) GSEA running enrichment score plot for “GO: Response to Xenobiotic Stimulus.” (**F**) GSEA running enrichment score plot for Hallmark pathway “Oxidative Phosphorylation.” (**G**) GSEA running enrichment score plot for Hallmark pathway “Fatty Acid Metabolism.” (**H**) Dot plot illustrating expression of zone 1, zone 2, and zone 3 gene expression modules in zonally ambiguous and stressed hepatocytes. Zone 1 genes: *FBP1*, *VTN*, *PIGR*, *ASS1*, *ARG1*, *PCK1*, and *SDS*. Zone 2 genes: *HAMP*, *TERT*, and *CCND1*. Zone 3 genes: *HMGCS2*, *CYP2E1*, *OAT*, *RGN*, *TBX3*, *AXIN2*, *CYP8B1*, and *COBLL1*.

From our cell type proportion analyses, a unique population of hepatocytes emerged and was enriched 4-fold in advanced FALD compared with both normal liver and early FALD (Figure 1C and Supplemental Figure 3B). To gain further insights, we investigated their unique transcriptomic profile to glean potential functional perspective. First, these hepatocytes in advanced FALD were enriched in markers of classical stress response pathways, characterized by increased expression of *HSPA1B*, *HSPH1*, *HSPA1A*, *JUN*, and *ATF3* (Supplemental Figure 4A). Hence, these hepatocytes were “stressed.” Furthermore, gene set enrichment analysis (GSEA) using Gene Ontology (GO) pathways comparing these cells to all other annotated cell types revealed significant enrichment of “GO: Response to Topologically Incorrect Protein” (normalized enrichment score [NES]: 2.66, *P* < 0.001) and ‘GO: Cellular Response to Unfolded Protein’ (NES: 2.44, *P* < 0.001) pathways (Figure 2A and Supplemental Figure 6, A and B). Additionally, GSEA using hallmark pathways comparing these unique “stressed hepatocytes” to all other annotated cell types revealed significant enrichment of “Hallmark: TNFA Signaling via NFKB” (NES: 2.01, *P* < 0.001) and “Hallmark: Reactive Oxygen Species” (NES: 1.56, *P* = 0.03) pathways (Supplemental Figure 6, C–E). Given the association between the unfolded protein response, inflammation, and oxidative stress with cellular senescence, we next investigated whether this hepatocyte subpopulation showed upregulation of various senescence markers (14). Interestingly, this subpopulation showed increased expression of *CDKN1A* and *SPI1* (Supplemental Figure 7A), along with increased expression of various senescence-associated secretory phenotype (SASP) markers, including *CXCL1*, *CXCL2*, *CXCL8*, *CCL4*, *GDF15*, *ICAM1*, *SERPINE1*, and *TIMP1* (Figure 2B and Supplemental Figure 7B), as compared with other hepatocyte subpopulations. As compared with healthy control and early FALD, *CXCL1*, *CXCL2*, *CXCL8*, *CCL4*, *GDF15*, *ICAM1*, and *TNF* were upregulated in advanced FALD (Supplemental Figure 7C). Next, to ascertain potential drivers of this senescence-like phenotype, we performed an upstream regulator analysis (Qiagen) on the differentially expressed genes (DEGs) (log_2_FC > 0.5, *P* < 0.05) from the “stressed hepatocyte” population, which identified that top kinases upstream of these genes were predicted to be MAP3K8, RAF1, MAPK3, IKBKB, and JAK1, which are all previously known to induce a senescence-like cellular state (Figure 2C) (15–18).

In normal liver, hepatocytes maintain a distinct pattern of compartmentalized gene expression where hepatocytes in distinct areas of the hepatic lobule perform specific functions, a phenomenon termed “liver zonation” (19). Specifically, hepatocytes in the periportal region (zone 1) perform key tasks such as gluconeogenesis, mitochondrial fatty acid β-oxidation, and ureagenesis, while hepatocytes in the pericentral region (zone 3) perform key tasks such as xenobiotic metabolism, bile acid metabolism, glycolysis, lipogenesis, peroxisomal fatty acid β-oxidation, and glutamine synthesis (20). In this manner, the liver is able to compartmentalize its functions and be efficient in performing multiple eclectic functions. Interestingly, in advanced FALD, the identified “stressed hepatocytes” were present across the entire lobule (Figure 1D). Thus, we hypothesized that given their pan-zonal distribution, these “stressed hepatocytes” likely dually express genes that are traditionally expressed solely in pericentral (zone 3) or periportal (zone 1) zones. Indeed, GSEA with GO and Hallmark pathways revealed significant enrichment of “GO: Bile Acid Biosynthetic Process” (NES: 1.87, *P* = 0.02), “GO: Glucose Metabolic Process” (NES: 1.80, *P* = 0.002), “GO: Response to Xenobiotic Stimulus” (NES: 1.44, *P* = 0.02), “Hallmark: Xenobiotic Metabolism” (NES: 2.31, *P* < 0.001), “Hallmark: Bile Acid Metabolism” (NES: 2.10, *P* < 0.001), and “Hallmark: MTORC1 Signaling” (NES: 1.38, *P* = 0.03) pathways, which are all canonical features of pericentral (zone 3) hepatocyte metabolism (20, 21). Additionally, GSEA with GO and Hallmark pathways revealed significant enrichment of “Hallmark: Oxidative Phosphorylation” (NES: 2.73, *P* < 0.001) and “Hallmark: Fatty Acid β-oxidation” (NES: 2.32, *P* < 0.001) pathways, which are canonical features of periportal (zone 1) hepatocyte metabolism (Figure 2, D–G). Additionally, gene expression module scores for zone 1 (*FBP1*, *VTN*, *PIGR*, *ASS1*, *ARG1*, *PCK1*, and *SDS*), zone 2 (*HAMP*, *TERT*, and *CCND1*), and zone 3 genes (*HMGCS2*, *CYP2E1*, *OAT*, *RGN*, *TBX3*, *AXIN2*, *CYP8B1*, and *COBLL1*) demonstrated enrichment of both zone 1 and zone 3 gene sets (Figure 2H). Overall, this analysis suggested these hepatocytes to be zonally ambiguous in their gene expression. Hence, we now annotate these hepatocytes that are both stressed and zonally misplaced in their gene expression profile and likely in their function as “zonally ambiguous and stressed hepatocytes” (Figure 2, D–G). The set of genes associated with these zonally ambiguous and stressed hepatocytes was upregulated as compared with other hepatocytes and parenchymal cell populations (Supplemental Figure 7D) and only observed in advanced FALD (Supplemental Figure 7E).

In summary, a hepatocyte population with both features of cellular stress and zonally mismatched gene expression is evident across the hepatic lobule in advanced FALD and may be contributing to hepatic dysfunction, representing maladaptation due to chronic insult evident in FALD disease progression.

### Liver zonation is disrupted in advanced FALD.

**Figure 3:**
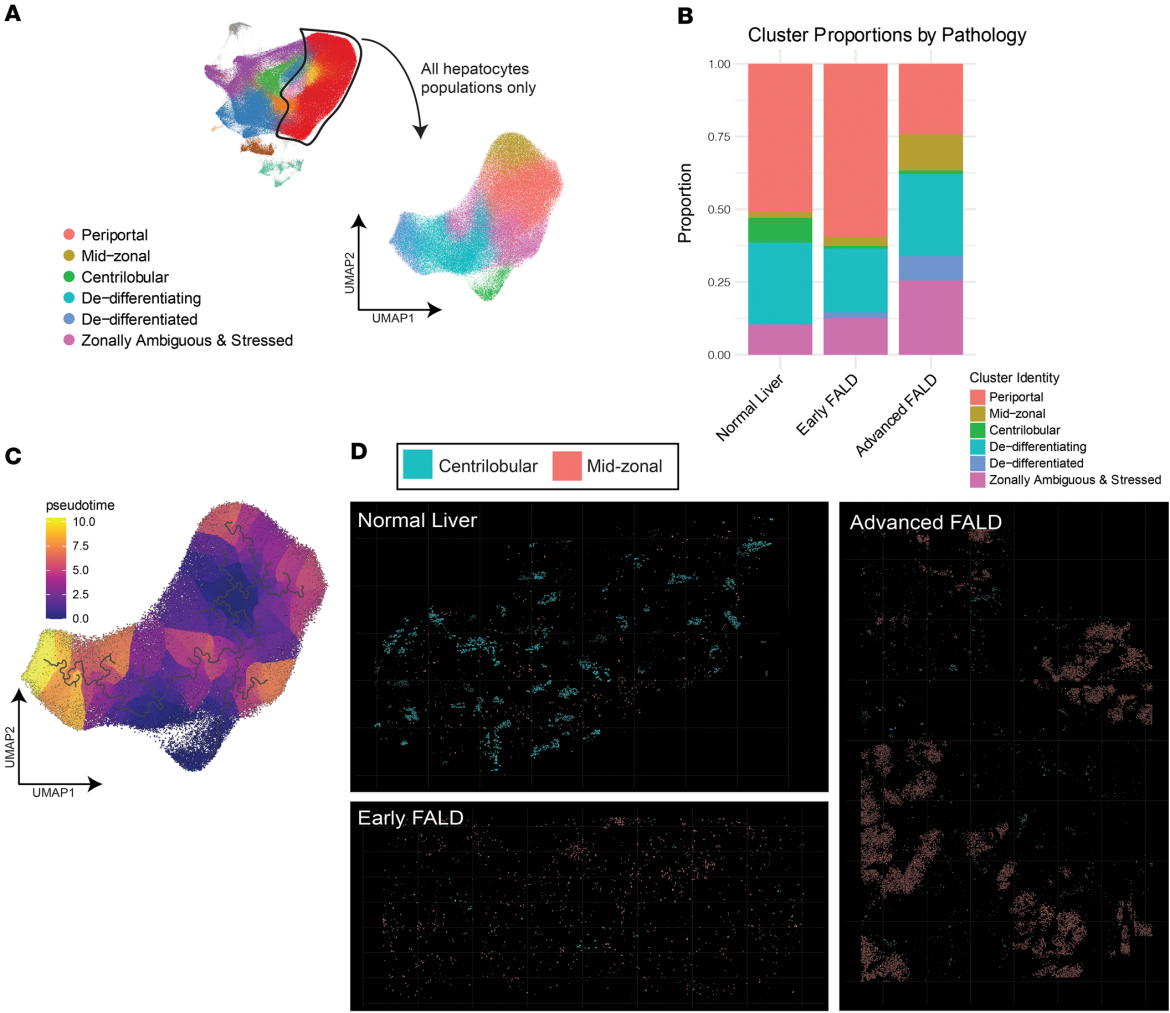
Liver hepatocyte zonation is disrupted in FALD. (**A**) Uniform manifold approximation and projection (UMAP) showing each hepatocyte subpopulation following subclustering of 3 hepatocyte populations: hepatocytes, centrilobular hepatocytes, and zonally ambiguous and stressed hepatocytes. (**B**) Stacked bar plot showing cluster proportions of hepatocyte subpopulations per disease pathology. (**C**) Pseudotime analysis of hepatocyte subpopulations in FALD. (**D**) Spatial plots of normal liver, FALD with early fibrosis, and FALD with advanced fibrosis for centrilobular and mid-zonal hepatocytes.

Next, to address hepatocyte gene expression and maladaptation more comprehensively in FALD, we performed further subclustering on the 3 original hepatocyte clusters, “centrilobular hepatocytes,” “zonally ambiguous and stressed hepatocytes,” and “hepatocytes,” identified in Figure 1B. We identified 6 unique hepatocyte populations consisting of periportal, mid-zonal, centrilobular, dedifferentiating, dedifferentiated, and zonally ambiguous and stressed hepatocytes (Figure 3A). Dedifferentiating and dedifferentiated hepatocytes were both defined by upregulation of pathways relating to epithelial-mesenchymal transition, Hedgehog signaling, and Notch signaling (Supplemental Figure 8, A and B), while dedifferentiated hepatocytes also uniquely expressed *VIM*, *LGR5*, *ACTL6A*, and *MCM5*, implying a more progenitor/stem-like hepatocyte phenotype, and concomitantly displaying downregulation of canonical zonation markers, *CYP2E1* and *ASS1* (Supplemental Figure 9) (22–24). Although the proportion of dedifferentiating hepatocytes was relatively stable across normal liver, early, and advanced FALD, the proportion of dedifferentiated hepatocytes increased in advanced FALD (Figure 3B). Additionally, pseudotime ordering revealed that centrilobular and periportal hepatocytes were the root node of the trajectory, with the lowest pseudotime values, reflecting their transcriptionally stable, mature, and functionally compartmentalized zonal states (Figure 3C). From these 2 endpoints, increasing pseudotime ordering captured a progressive loss of hepatic zonal identity, with emergence of a stress-adaptive, zonally ambiguous transcriptional program along the trajectory (Figure 3C). This continuum ultimately converged on dedifferentiating and fully dedifferentiated hepatocyte populations, which had the highest pseudotime values, minimal hepatocyte zonal identity, and upregulation of a progenitor/stem-like gene program (Figure 3C). Overall, this observation suggests that the 2 mature zonal hepatocyte states dedifferentiate toward a progenitor-like endpoint as part of maladaptive response during chronic liver injury.

Interestingly, a key difference in overall hepatocyte zonation between early and advanced FALD was the expansion of mid-zonal (zone 2) hepatocytes, indicated by high expression of *HAMP* in this cell population (Figure 3B and Supplemental Figure 9A). In a normal liver, mid-zonal hepatocytes were represented by a few layers in between the central vein and the portal vein (Figure 3D). This was largely recapitulated in early FALD as well. However, in advanced FALD, mid-zonal hepatocytes expanded (Figure 3D), likely in response to chronic liver injury, as these hepatocytes are known to maintain liver homeostasis (25). Overall, as FALD progressed, we observed a notable change in hepatocyte zonal gene expression across the entire hepatic lobule, evidenced by loss of periportal and centrilobular hepatocytes, and appearance of de novo clusters including the zonally ambiguous and stressed hepatocytes and the expansion of mid-zonal hepatocytes. While it is unclear whether these adaptations are compensatory, and thus an attempt to maintain function, elucidating cellular and molecular basis driving these changes is highly relevant.

### Endothelial cell zonation is disrupted in advanced FALD.

**Figure 4:**
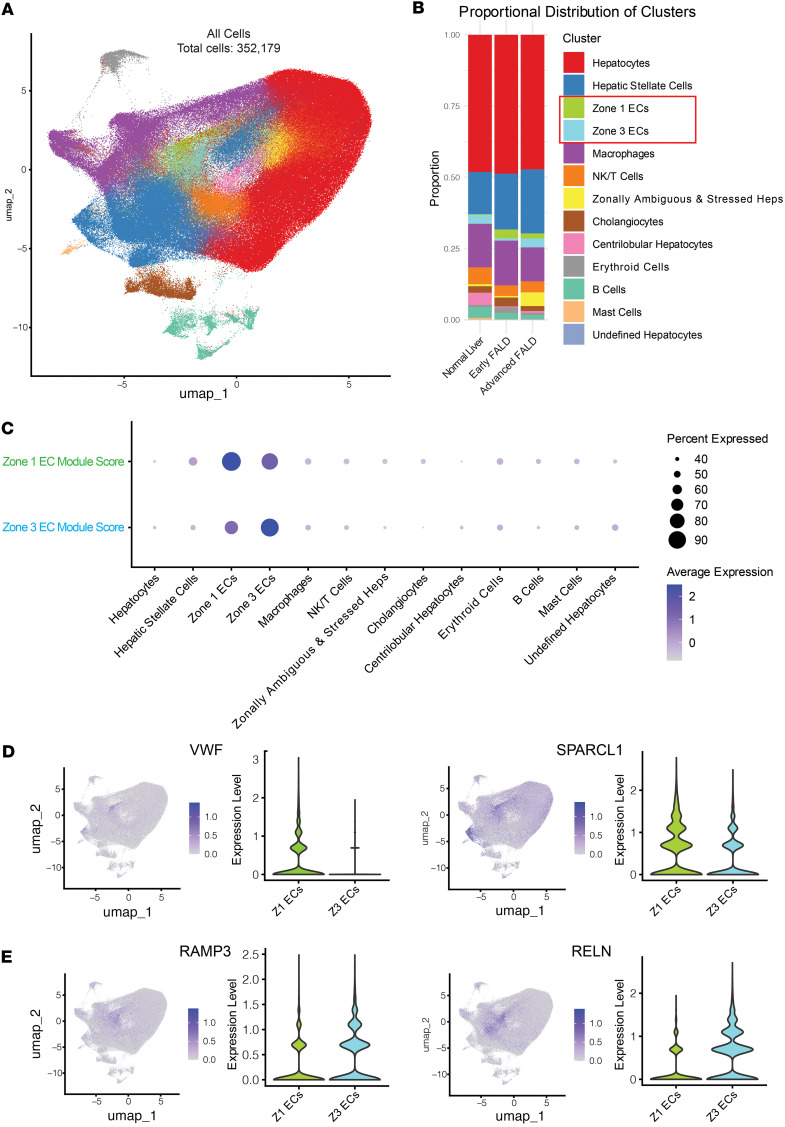
Liver endothelial cell zonation is disrupted in FALD. (**A**) Uniform manifold approximation and projection (UMAP) showing zone 1 and zone 3 endothelial cells. (**B**) Stacked bar plot showing relative proportions of zone 1 and zone 3 endothelial cells in FALD. (**C**) Dot plot showing expression of zone 1 and zone 3 endothelial cell gene expression module scores across the different cell types (module score: using the normal liver cell atlas, top 25 differentially expressed genes were selected for each endothelial cell subtype). (**D**) Feature and violin plots of zone 1 endothelial cells marker genes. (**E**) Feature and violin plots of zone 3 endothelial cells marker genes.

Given the pan-zonal distribution of zonally ambiguous and stressed hepatocyte subpopulation, we were next interested in determining how these cells physically located in zone 1 were exhibiting gene expression (and in turn pathways) that are normally evident in zone 3 hepatocytes. Compared with zones 1 and 2, the major driver of zone 3 hepatocyte gene expression and function has been carefully dissected with a fundamental role of the Wnt/β-catenin signaling in this process (20). In fact, Wnts secreted from zone 3 endothelial cells, specifically Wnt2 and Wnt9b, directly control zonation via paracrine β-catenin activation (19). Knowing the key relevance of endothelial cells in the process of hepatocyte gene expression, especially in zone 3, we next subclustered the endothelial cells into zone 1 and zone 3 subpopulations using previously published marker genes (13). We then annotated these cell types in the larger CosMx dataset (Figure 4, A–C), based on gene expression module scores for zone 1 and zone 3 endothelial cells. Zone 1 endothelial cells showed increased expression of *VWF* and *SPARCL1*, while zone 3 endothelial cells showed increased expression of *RAMP3* and *RELN*, which are known markers for each of these subpopulations (Figure 4, D and E) (26). Next, we performed GSEA with GO and Hallmark pathway sets for zone 1 and zone 3 endothelial cells to infer their biological significance. Interestingly, despite both endothelial cell populations demonstrating upregulation of the hypoxia pathways, zone 1 endothelial cells demonstrated upregulation of endothelial cell migration pathways (Supplemental Figure 10, A–D). Furthermore, to corroborate this, spatial plots of centrilobular hepatocytes and zone 1 and 3 endothelial cells demonstrated that in normal liver, zone 3 endothelial cells surround the central veins, while in FALD with advanced fibrosis, zone 1 endothelial cells expanded and lacked a clearly defined zonation pattern (Supplemental Figure 11A). Overall, in advanced FALD, endothelial cells lose their spatial patterning.

### Ectopic WNT2 from periportal endothelial cells facilitates zone 3 hepatocyte gene expression across the hepatic lobule in FALD.

**Figure 5:**
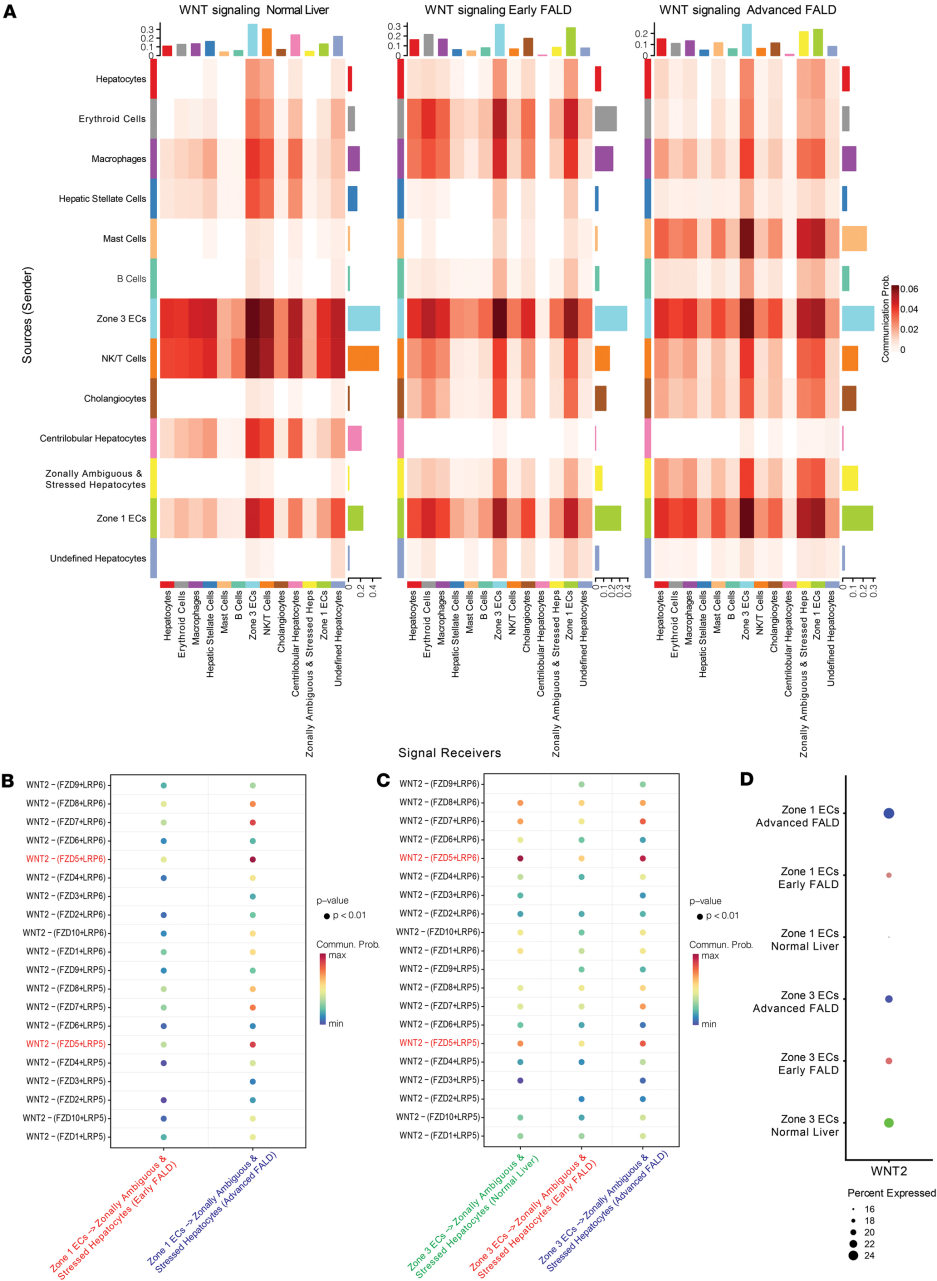
Periportal (zone 1) endothelial cell to zonally ambiguous and stressed hepatocyte crosstalk via WNT2/FZD5/LRP5/6 axis identified via CellChat. (**A**) Heatmap of cellular communication for WNT signaling by disease pathology. (**B**) Bubble plot of WNT/FZD signaling between zone 1 endothelial cells and zonally ambiguous and stressed hepatocytes across disease pathology. (**C**) Bubble plot of WNT/FZD signaling between zone 3 endothelial cells and zonally ambiguous and stressed hepatocytes across disease pathology. (**D**) Dot plot of WNT2 across zone 1 and zone 3 endothelial cells and disease pathology in the CosMx dataset.

Next, we hypothesized that cell-to-cell Wnt signaling was likely altered due to both an observed increase in zone 1 endothelial cells in the pericentral region, along with the presence of zonally ambiguous and stressed hepatocytes expressing zone 3 (pericentral) genes near zone 1 (or the periportal region). Thus, using CellChat (27) analysis on the CosMx dataset, we investigated global changes in Wnt signaling in FALD. Interestingly, across the entire dataset, endothelial cells (both zone 1 and zone 3) had the highest communication probability of Wnt signals to the zonally ambiguous and stressed hepatocytes (Supplemental Figure 12A). Additionally, when segregating out the cell-to-cell communication by disease pathology, zone 1 endothelial cell Wnt signals showed increased communication probability from normal liver to advanced FALD (Figure 5A). Next, using CellChat, we investigated which *WNT-FZD/LRP* interaction had the highest probability for communication. Across the entire dataset, *WNT3A*, *WNT4*, and *WNT2* showed the highest relative contribution among all WNTs (Supplemental Figure 13A). Of these 3 *WNTs, WNT2* (along with *WNT9B*) from the zone 3 endothelial cells has been conclusively identified as facilitating zone 3 (pericentral) hepatocyte gene expression and downstream functions (19). Interestingly, *WNT2* expression was observed from both zone 3 and zone 1 endothelial cells across the entire dataset in advanced FALD (Supplemental Figure 13B). In normal liver, *WNT2* is only expressed in zone 3 endothelial cells, but in FALD, *WNT2* expression originates from zone 1 endothelial cells as well (Supplemental Figure 12, B and C). Further analysis revealed that *WNT2* from both zone 1 and zone 3 endothelial cells communicates with zonally ambiguous and stressed hepatocytes via FZD5/LRP5/6 (Figure 5, B and C). Lastly, we quantified this *WNT2* expression increase from zone 1 endothelial cells and observed an increased gradient of *WNT2* expression from zone 1 endothelial cells in normal liver to FALD with early and advanced fibrosis (Figure 5D and Supplemental Figure 13C). Thus, we identified a putative cell signaling circuit whereby ectopic expression of WNT2 from zone 1 endothelial cells facilitates pericentral hepatocyte gene expression (and likely downstream functions) in zonally ambiguous and stressed hepatocytes within the periportal region via a WNT2/FZD5/LRP5/6 axis.

### Zonally ambiguous and stressed hepatocytes are enriched in end-stage liver disease.

Lastly, we were interested in whether our findings were unique to FALD or shared among other progressive and chronic liver diseases. We analyzed a previously published single-nucleus RNA-seq dataset (GSE202379), which contained single-cell transcriptomes from normal adult liver, metabolic dysfunction–associated steatotic liver disease (MASLD), MASH with and without cirrhosis, and end-stage liver disease (Supplemental Figure 14, A and B). Interestingly, a subset of hepatocytes in end-stage liver disease patients had increased expression of the zonally ambiguous and stressed hepatocyte marker genes *HSPA1B*, *HSPH1*, *HSPA1A*, *JUN*, *FOS*, and *ATF3* (Supplemental Figure 14C). Moreover, end-stage liver disease patients had increased expression of a gene module score based on DEGs from zonally ambiguous and stressed hepatocytes (Supplemental Figure 14D). The transcriptomics dataset also demonstrated a similar increase in HSP70 module score expression in end-stage liver disease, without significant upregulation in other samples of MASLD and MASH (Supplemental Figure 14D). While the HSP70 module score and contributing subunits were upregulated, albeit to a lesser degree in the healthy control samples (Supplemental Figure 14E), we are limited in providing a clinical correlation to this upregulation, as the study did not provide a clinical description of the “healthy control specimens.” Moreover, analogous to our endothelial cell analysis in FALD (Figure 4A), we subclustered the endothelial cells in this MASLD/MASH dataset as well. Similarly, we observed *WNT2* upregulation in zone 1 endothelial cells in end-stage liver disease patients compared with healthy controls (Supplemental Figure 15, A and B), thereby indicating a significant overlap in pathophysiology at the end stage of multiple etiologies of metabolic liver diseases.

Thus, zonally ambiguous and stressed hepatocytes may be a feature of end-stage liver disease shared across multiple etiologies, but may be driven by mechanisms distinct to each underlying disease process.

### HSP70 correlated with the CHF score in FALD.

**Figure 6:**
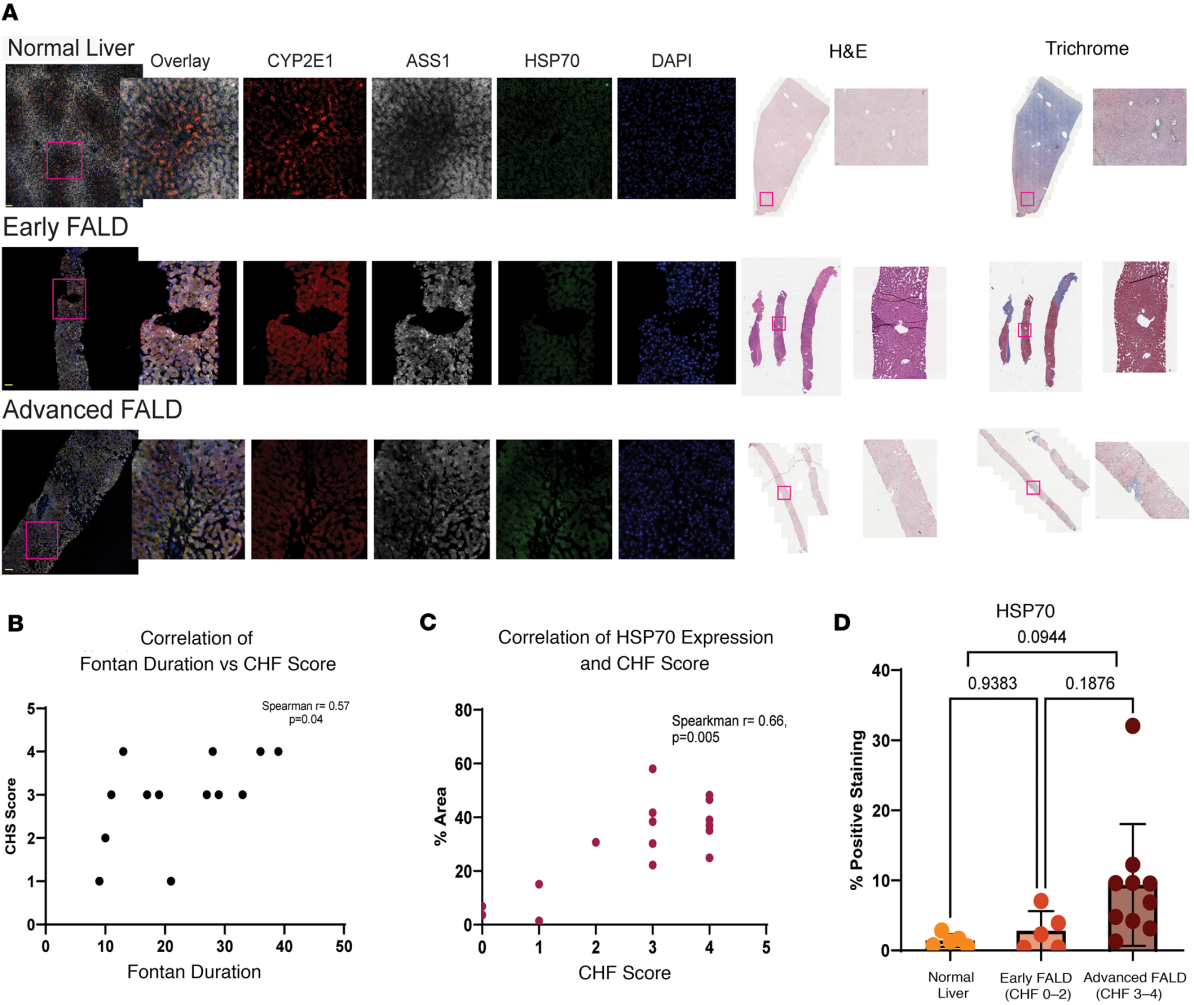
Immunofluorescence of hepatocyte zonation markers and HSP70 demonstrates altered zonation and increased cellular stress in advanced FALD. (**A**) Normal liver sample: Liver tissue obtained from a healthy control shows pericentral distribution of zone 3 marker CYP2E1, periportal distribution of ASS1, and minimal expression of HSP70 in liver parenchyma. Early FALD: Liver biopsy tissue obtained from a Fontan patient with a CHF score of 0 shows relatively preserved hepatic lobule architecture, but expansion of zone 1 and zone 3 markers as well as an increase of pericentral HSP70 expression. Advanced FALD: Liver biopsy tissue obtained from a patient with CHF score of 3, shows abnormal zonation with unorganized CYP2E1 and ASS1 distribution and an increase in HSP70 expression in liver parenchyma. Scale bar: 7.5 μm. Inset: ×200. H&E and trichrome: ×200. (**B**) Congestive hepatic fibrosis (CHF) score correlation with Fontan duration in FALD patients. (**C**) CHF score correlation with HSP70 expression in FALD patients. (**D**) Percentage positive HSP70 staining by disease pathology. Differences between groups (*P* values shown on top) were assessed using 1-way ANOVA followed by Tukey’s multiple-comparison test.

Immunofluorescent staining of zonation markers CYP2E1 (zone 3) and ASS1 (zone 1) confirmed the disruption of zonation at a protein level, as indicated by our spatial transcriptomic data (Figure 6, A and B, and Supplemental Figure 16). The CHF score was used to determine the extent of fibrosis in FALD clinical samples stained with H&E and trichrome staining, as this has been previously validated in FALD (28). Samples with CHF scores of 0–2 were classified as early FALD according to the following: 0, no fibrosis; 1, central zone fibrosis; 2A, central zone and mild portal fibrosis with accentuation at central zone; 2B, at least moderate portal fibrosis and central zone fibrosis, with accentuation at portal zone. CHF scores of 3 and 4 were classified as advanced FALD, where 3 indicates bridging fibrosis and 4 cirrhosis. Uniquely, a liver biopsy specimen obtained from a FALD patient with a CHF score of 0 showed relatively preserved hepatic lobule architecture, with minimal expansion of zone 1 and zone 3 markers. The majority of liver biopsy specimens obtained from FALD patients with CHF scores 1–4 showed abnormal zonation with unorganized CYP2E1 (zone 3) and ASS1 (zone 1) distribution. Interestingly, advanced FALD samples (CHF scores 3 and 4) had a significant increase in HSP70 expression. Interestingly, in FALD, HSP70 was expressed within the hepatic parenchyma, as compared with localization primarily to bile ducts in normal liver specimens (Figure 6, A and B). Indeed, correlational analysis showed that CHF score positively correlated with time of Fontan duration (Spearman’s *r* = 0.57, *P* = 0.04) (Figure 6B). Lastly, CHF score strongly correlated with HSP70 tissue area expression (Spearman’s *r* = 0.66, *P* = 0.005) (Figure 6C) and a trend toward increased HSP70 expression with disease pathology (early vs. advanced FALD; *P* = 0.09) (Figure 6D).

## Discussion

We established the first single-cell spatial transcriptomic atlas to our knowledge of early and advanced fibrosis in human FALD. The 2015 proceedings from the American College of Cardiology stakeholders meeting summarized that concerted efforts are essential for further understanding of the “risk factors, pathophysiology, longitudinal consequences, and therapeutic options related to FALD” (9). Here, we described diseased cell-type-specific transcriptional changes, along with the spatial location of these parenchymal and non-parenchymal hepatic cell types in human FALD with early and advanced fibrosis. First, we identified that liver zonation was disrupted in FALD. Specifically, as FALD progressed, we observed an expansion of mid-zonal hepatocytes, which likely contributed to the maintenance of liver homeostasis secondary to the loss of mature periportal and pericentral hepatocytes. We also identified what we believe is a unique hepatocyte subpopulation, which we have termed “zonally ambiguous and stressed hepatocytes,” which is also found in end-stage liver disease of other etiologies. This cell population expressed cellular stress markers and exhibited aberrant zonation, characterized by the simultaneous expression of centrilobular and periportal genes. Additionally, we revealed that aberrant endothelial cell zonation likely facilitated altered hepatocyte zonation. Specifically, zonally ambiguous and stressed hepatocytes residing in the periportal (zone 1) region expressed pericentral (zone 3) genes through ectopic *WNT2* expression from zone 1 endothelial cells. Overall, we identified a cell-to-cell signaling axis that helps to maintain liver homeostasis as part of a maladaptive response during chronic liver injury in FALD.

The cellular remodeling that occurs in FALD remains poorly understood. Our spatial mapping demonstrated that in early FALD, centrilobular hepatocytes begin to lose their identity, with a complete loss of this cell type as the disease progressed. A recent single-nucleus RNA-seq study by Hu et al. of human FALD with early fibrosis similarly revealed that centrilobular hepatocytes underwent the most profound transcriptional changes, which occurred prior to HSC activation and fibrosis (29). In this analysis, centrilobular hepatocytes were shown to upregulate key genes involved in bile acid metabolism, which we observed here as well (30). With sequential loss of centrilobular hepatocytes during disease progression, there is a disruption of bile acid metabolism, leading to an increase in the total bile acid pool, likely explaining the development of cholestasis (31, 32). Interestingly, Kogiso et al. tested whether ursodeoxycholic acid (UDCA), which helps improve bile acid secretion, would delay disease progression in FALD (33). Remarkably, FALD patients with UDCA treatment had a lower incidence of hepatocellular carcinoma development than patients not treated (5.6% vs. 24.2%) (33). Thus, our findings in conjunction with prior reports have elucidated a likely cellular explanation for the relatively improved outcomes of FALD patients with UDCA treatment. Future studies should aim to test bile acid modulation as a potential therapeutic target in FALD to prevent disease progression, due to the likely effect on preserving centrilobular hepatocyte function.

The majority of FALD patients undergo non-invasive surveillance, including serum and imaging studies, which limits the available tissue for study. To assess the degree of hepatic injury, function, and portal hypertension, ultrasound imaging is not the most informative assay, and serum liver function tests tend to be mildly elevated and do not correlate well with disease severity (34). However, despite tissue biopsy being the current gold-standard approach for assessing the degree of hepatic fibrosis, biopsies are not routinely pursued due to a higher clinical risk of complications, as well as the risk of sampling bias resulting from FALD, which can lead to focal areas of liver fibrosis (34). Thus, there is a critical need to define clinically relevant non-invasive biomarkers that can differentiate patients with early and advanced fibrosis in FALD. It is equally important to understand the cellular source associated with such a proposed biomarker. Interestingly, one of the top upregulated genes in our zonally ambiguous and stressed hepatocyte subpopulation was *GDF15*, which we have previously shown to independently correlate with clinical progression in FALD patients (35). We have now identified a possible predominant source of GDF15 localized to zonally ambiguous and stressed hepatocytes, rather than the proposed origin in HSCs. Intriguingly, various studies in chronic liver disease have elucidated a protective role of hepatocyte-derived GDF15 in preventing liver inflammation and fibrosis (36). In MASLD/MASH, senescent hepatocyte burden correlates with GDF15 expression in circulation, and senolytics improves fibrosis in small animal models (37). Thus, future studies are needed to determine the role of hepatocyte-derived GDF15 in FALD as well as its systemic effect on subsequent cardiac function, as different cellular sources and mechanisms of liver injury likely mediate distinct aspects of disease pathogenesis.

By utilizing single-cell spatial transcriptomics (ST), we have been able to demonstrate that specific cell types reside within key cellular niches in FALD, both in early and advanced disease states. Of particular interest here is the zonally ambiguous and stressed hepatocyte subpopulation, which is a distinct cell population that differentiates early and advanced fibrosis in FALD. In fact, a bulk transcriptomic study by Bravo-Jaimes et al. on 106 FALD patients with early and advanced fibrosis revealed that the top upregulated genes in patients with advanced versus early fibrosis were *HSPA1A, JUN*, and *FOSB*, which were the equivalent upregulated genes unique to our zonally ambiguous and stressed hepatocyte population (11). Here, we not only provide a cellular source for these gene expression changes observed in advanced FALD, but also demonstrate that this cell population resides in close proximity to both zone 1 and zone 3 endothelial cells, reflecting the zonally ambiguous gene expression program. Over time, these paracrine Wnt signals likely drive this hepatocyte phenotype across the hepatic lobule. Moreover, other chronic liver disease pathologies, including MASLD and MASH, have shown a similar loss of zonation and ambiguous zonal patterning, and additional analysis of these samples confirms the upregulation of ectopic Wnt signaling in end-stage liver disease (38). Future in vitro and in vivo studies aimed at identifying upstream regulators of zonal reprogramming of hepatocytes, driven by disrupted endothelial cell zonation, whether due to mechanical stress leading to increased central venous pressures or chronic hypoxia, will lead to potential therapeutic targets for FALD patients. The presence of these cells across other liver pathologies, such as MASLD/MASH, is reassuring and can provide therapeutic insights for reversing these pathologic cellular phenotypes.

It is likely that in the zonally ambiguous and stressed hepatocyte population, expression of otherwise compartmentalized genetic programs into this one hepatocyte population, induces a state of cellular stress and senescence-like characteristics. First, we observed upregulation of the heat-shock pathway with expression of multiple genes in the *HSP70* family, namely *HSPA1A*, *HSPA1B*, and others. Various HSPs have been shown to have both profibrotic and antifibrotic roles in chronic liver disease through regulation of extracellular matrix, depending on the disease context (39). Specifically, HSP70 has been shown to induce proinflammatory cytokines that are integral during early liver regeneration through upregulation of TNF-α (40). HSP70 has also been shown to correlate with hepatic dysfunction and disease severity in postoperative biliary atresia (41). Similarly, HSP70 can serve as a clinical marker to determine the degree of cirrhosis for considering transplantation in Fontan patients. The zonally ambiguous and stressed hepatocytes also showed a significant increase in the senescence-associated genes *CDKN1A* (protein p21) and *SPI1* (transcription factor PU.1). CDKN1A is involved in stabilizing cell cycle arrest following senescence induction and is associated with MASLD/MASH disease severity, fibrosis, and degree of inflammation in MASLD/MASH (42, 43). Similarly, SPI1 is expressed in multiple parenchymal and non-parenchymal cell types in the liver and although its role in the liver is less studied, it has been shown to induce stem cell quiescence and limit expansion during stress states (44). Whether the emergence of the zonally ambiguous and stressed hepatocyte population is a cause or a response to increased demand to consolidate multiple gene expression programs once held by zonally resident hepatocytes will require future studies investigating disease initiation and progression in preclinical models.

Lastly, the role of endothelial cell zonation driving hepatocyte zonation is also integral in preserving homeostasis in the liver. As pericentral (zone 3) endothelial cells are in close proximity to the body’s most hypoxic blood from central venous circulation, as well as chronically high pressures due to the Fontan physiology, our data suggest either a loss of these cells from the pericentral region via migration, or reprogramming of their identity. Indeed, endothelial cell zonation, and upregulation of zone 3–specific WNTs (i.e., WNT2) in zone 1 endothelial cells provide a unique insight into liver plasticity. Future studies in understanding the systemic effects related to this zonal reprogramming of the liver are essential for understanding the long-term effects of Fontan physiology and better management of our Fontan patients.

There are a few limitations worth noting in this study. First, we used 1 early and 1 advanced FALD sample for the initial discovery phase of cellular phenotypes using spatial transcriptomics, which is a small sample size. However, we further validate our key findings of altered zonation and emergence of the zonally ambiguous and stressed hepatocyte subpopulation using immunofluorescence in 18 additional FALD samples, increasing the rigor of our findings. Second, the liver samples used for our spatial transcriptomics were very different in age (early FALD: 59 years and advanced FALD: 21 years). Since the older patient has early FALD, our findings underscore that biological factors primarily drive FALD progression. Further validation with other FALD patients and age-matched controls is warranted to expand upon our initial findings presented throughout. Lastly, although the CosMx SMI platform provides high spatial resolution, cell type clustering is performed using a targeted gene panel of approximately 6000 genes, which when contrasted with single-cell RNA-seq, is more limited in the ability to fully characterize all parenchymal and non-parenchymal cell states in the liver. Future work will aim to integrate both platforms together to appreciate full resolution of various cell states during FALD progression.

Overall, our single-cell spatial transcriptomic atlas of FALD with early and advanced fibrosis identified a unique hepatocyte subpopulation and spatial cellular niches of parenchymal and non-parenchymal cells defining advanced FALD pathobiology. Advanced FALD was defined by the expansion of mid-zonal hepatocytes with the loss of canonical pericentral and periportal hepatocytes. As a result of chronic congestion, likely inducing mechanical stretch and hypoxia, liver zonation was perturbed through what we believe is a unique paracrine signaling axis, which reprogrammed distinct hepatocyte subpopulations. Over time, the increased demand in a subset of hepatocytes to perform multiple functions induces cellular stress and a senescence-like state, which we refer to as zonally ambiguous and stressed hepatocytes. Future work aims to define non-invasive biomarkers reflecting the origin of zonally ambiguous and stressed hepatocytes, as these may help stratify FALD patients who need liver transplantation.

## Methods

### Sex as a biological variable

The FALD samples were obtained from both male and female patients. Given the small number of FALD samples, sex was not considered as a biological variable in our outcomes.

### Tissue explant/biopsy specimens and associated clinical data

Fontan patients with liver biopsy or explanted liver tissue in the past 20 years (2005–2025) at the Children’s Hospital of Pittsburgh and the UPMC were identified, and formalin-fixed paraffin-embedded (FFPE) blocks were retrieved. Preperfusion normal liver allografts were used as controls. Blocks with adequate tissue were sectioned as described below. Related clinical information, including the patient’s age at the time of tissue retrieval and the duration of Fontan completion, was obtained through a chart review conducted by the exempt Internal Review Board of the University of Pittsburgh.

### SMI of FFPE liver tissues using CosMx

FFPE liver tissue samples were analyzed using the CosMx SMI platform from NanoString Technologies, a Bruker company, at the Center for Computational Immunogenetics of the University of Pittsburgh. Sample preparation was performed according to protocol MAN-10184-02 using the Human Universal Cell Characterization 6K Plex Panel (part number 121500041). Tissue sections were cut at 5 μm thickness, mounted on Superfrost Plus Micro Slides, and baked overnight at 60°C to enhance adherence. After deparaffinization, target retrieval was performed at 95°C–100°C for 15 minutes, followed by permeabilization with 3 μg/mL Proteinase K at 40°C for 30 minutes. Fiducial markers were applied for precise image alignment, followed by postfixation with neutral buffered formalin and blocking with *N*-hydroxy succinimide (NHS)–acetate. The slides were then hybridized overnight at 37°C with RNA-specific probes from the 6K RNA panel. Following stringent washes to remove unbound probes, DAPI staining was used for nuclear visualization, and cell segmentation markers CD298 and β-2-microglobulin (B2M), along with additional cellular markers PanCK and CD45, were applied. The prepared slides were loaded into the CosMx SMI for imaging. During imaging, branched fluorescent probes were hybridized to amplify signals, enabling the detection of 6000 RNA targets within individual cells. The raw images were processed and decoded using the AtoMx Spatial Informatics Platform (SIP) (https://brukerspatialbiology.com/products/atomx-spatial-informatics-platform/atomx-sip-overview/), a cloud-based service that provided the data for visualization and detailed analysis.

### Bioinformatic analysis

#### Data preprocessing and quality control.

The sequenced data was visualized in the AtoMx SIP. The initial data preprocessing was performed in their cloud-based software, utilizing a standard pipeline to ensure data quality control. The preprocessed data were subsequently exported from the AtoMx SIP as a Seurat object. The RDS file object was imported into R software version 4.4.0 (https://cran.rstudio.com/), which was used with the Seurat package (45) version 5.1.0 for data analysis and visualization. Each tissue section was first analyzed independently for data quality control. Inspection of data quality was first performed by analyzing the nCount_RNA (total number of molecules or reads across all genes in each cell) and nFeature_RNA (number of unique genes in each cell) on a histogram and violin plot from the Seurat object. The images and all fields of view (FOVs) were visualized to ensure adequate tissue sectioning before sequencing. Gene expression of known landmark genes was visualized at this step. Low-quality data were filtered out at this step (nCount_RNA < 5 and nFeature_RNA < 4 were excluded; Supplemental Figure 2, D and E). Following this step, the multiple Seurat objects were merged together with the merge() function and catalogued with unique cell identifications to group by at a later step in the analysis. SCTransform() (46) was performed on the merged Seurat object for data normalization and scaling, followed by principal component analysis on 3000 variable features via the RunPCA() function and uniform manifold approximation and projection (UMAP) via the RunUMAP() function with dims set to 1:30. The DimPlot() function was utilized to visualize the UMAP prior to data integration.

#### Data integration and dimensionality reduction analysis.

Next, data integration was performed to align shared cell types and states across the different tissue specimens and allow accurate comparison. Seurat v5 has a standard pipeline for data integration with SCTransform-normalized datasets (https://satijalab.org/seurat/articles/integration_introduction). The IntegrateLayers() function was subsequently used to perform data integration with the method set to “Harmony” (12) and normalization.method set to “SCT” since we performed SCTransform normalization. The FindNeighbors() function was set to dims=1:30 with reduction=”harmony”. The FindClusters() function was set with resolution=0.5. RunUMAP() function was set to dims=1:30 and reduction=”harmony”.

#### Data visualization, differential gene expression, and cell cluster annotation.

Visualization of projected cell clusters was performed using the DimPlot() and FeaturePlot() functions in Seurat. Spatial visualization of the indicated cell clusters onto the tissue section and gene expression of specific genes was performed using ImageDimPlot() and ImageFeaturePlot() functions, respectively, in Seurat. The indicated FOVs were specified in each Image function. The PrepSCTFindMarkers() function was used to prepare the Seurat object with multiple SCT models for differential expression analysis. The FindAllMarkers() function was used to identify DEGs in each of the cell clusters identified based on the resolution parameter set in the FindClusters() function. Genes were considered significant if the adjusted *P* value was less than 0.05. Cell clusters were then combined based on shared identity using previously published marker genes for different cell types (13). These new identities were then stored in the merged Seurat object. Differential gene expression for each of the new identities (annotated cell types) was performed using the FindMarkers() function using the non-parametric Wilcoxon rank-sum test. The adjusted *P* value was based on the Bonferroni correction. Top DEGs were then visualized via volcano plots with the EnhancedVolcano package.

### GSEA

Pathway GSEA was performed using DEGs identified by the FindMarkers() function. All genes were used for GSEA ranked by log_2_FC values in descending order. The fgsea package (47) was used for GSEA analysis. Hallmark and GO pathways were downloaded from the Molecular Signatures Database (MSigDB) (48, 49) and loaded into R. In the fgsea() function, minsize=5 and maxsize=1000 were used. Waterfall plots from the ggplot package were utilized to rank the top 10 and bottom 10 pathways by NES and *P* value. A GSEA running enrichment score plot was used to visualize enrichment of the gene set in the data.

### Cellular communication analysis via CellChat

Cellular communication analysis based on ligand-receptor expression was performed using the CellChat package (27) in R. We utilized the base pipeline from the CellChat vignette documentation. Cellular communication was depicted with bubble plots, hierarchy plots, circle plots, heatmaps, and chord diagrams, which were part of the visualization tools in the package.

### Public data mining of single-cell and spatial datasets

To determine expression of specific genes across cell types in normal adult liver, MASLD, MASH, and end-stage liver disease, we utilized the single-nucleus RNA-seq dataset previously deposited (GSE202379) (38). The processed and integrated dataset was directly downloaded from the NCBI Gene Expression Omnibus (GEO) as a Seurat RDS object and loaded into R. FeaturePlot() and VlnPlot() functions were utilized to visualize gene expression from particular cell populations. To define a signature of zonally ambiguous and stressed hepatocytes, the AddModuleScore() function was used to calculate a feature score based on the expression level of a list of the top DEGs identified from the zonally ambiguous and stressed hepatocyte subpopulation (*HSPA1B*, *JUN*, *HSPH1*, *HSPA1A*, *ATF3*, *FOS*, *BAG3*, *FST*, *CXCL2*, *DNAJB1*, and *GDF15*) in the CosMx dataset.

### Immunofluorescent staining on FFPE tissue

Tissue sections (4–6 μm) were processed for immunofluorescent staining following deparaffinization and heat-induced antigen retrieval with a pressure cooker for 20 minutes (citrate buffer pH 6). Primary antibodies (ASS1: Abcam, ab77590, 1:100; Cyp2e1: Sigma-Aldrich, HPA009128, 1:100; HSP70: Santa Cruz Biotechnology, sc-32239, 1:50) were incubated overnight at 4°C in a humidified chamber before incubation with fluorescently labeled secondary antibodies (Cy5: Jackson ImmunoResearch, 705-175-147; Thermo Fisher Scientific, NC9815052 and NC0254454, 1:200) for 1 hour at room temperature. Slides were scanned with a Nikon Eclipse Ti fluorescence microscope at ×20 magnification. Positive pixels (FITC-positive or Alexa Fluor 488–positive) were selected from each image and measured using ImageJ (NIH). Positive pixels were divided by the total number of pixels in the image to calculate the percentage positive HSP70 staining.

### Statistics

Statistical methods used for the analysis of single-cell spatial transcriptomic data are described in the Methods section. Differences in the percentage of tissue expressing HSP70 among WT, early FALD, and late FALD groups were assessed using 1-way analysis of variance (ANOVA). Post hoc pairwise comparisons were performed using Tukey’s multiple-comparison test. A *P* value of 0.05 or less was considered significant.

### Study approval

The study was reviewed and approved by the University of Pittsburgh Internal Review Board Pittsburgh, protocol number 19050089. Since these patient samples were obtained retrospectively from excess tissue after pathologists had provided a clinical diagnosis, patient-informed consent was not obtained.

### Data availability

Single-cell spatial transcriptomic data generated for this study are available on the GEO database with accession number GSE310443. The data points used to generate the graphs are available in the Supporting Data Values file.

## Author contributions

Designing research studies: BML, JNL, SPM, and AS. Conducting experiments: BML, JNL, and VM. Acquiring data: BML, MBN, LS, CKG, and AS. Analyzing data: BML, JJL, and SL. Providing reagents: AS, VOM, RS, and SPM. Writing the manuscript: BML, SPM, and AS.

## Conflict of interest

The authors have declared that no conflict of interest exists.

## Funding support

This work is the result of NIH funding, in whole or in part, and is subject to the NIH Public Access Policy. Through acceptance of this federal funding, the NIH has been given a right to make the work publicly available in PubMed Central.

- NIH grants T32EB001026 and F30CA284540 (to BML).
- NIH grant K08 HL 161440 (to AS).
- American Heart Association CDA 852875 (to AS).
- NIH grants R01DK103775 and R01DK062277 (to SPM).
- NIH grant P30DK120531 (to the Genomics and Systems Biology Core of the Pittsburgh Liver Research Center).
- NIH grant S10OD028483 (to the University of Pittsburgh High-Throughput Computing cluster.
- NIH grant S10MH126905 (to the University of Pittsburgh Center for Biologic Imaging).

## Supplementary Material

Supplemental data

## Version 1 02/24/2026

In-Press Preview

## Version 2 07/08/2026

Electronic publication
