# Supplementary material for: Single-cell spatial transcriptomics reveals hepatocyte reprogramming in Fontan-associated liver disease
Source: JCI Insight. 2026 Feb 24;11(13):e198823. doi: 10.1172/jci.insight.198823 (PMC13460813; doi:10.1172/jci.insight.198823)
Supplement: Supplementary file 1 [file jciinsight-11-198823-s205.pdf]

Supplementary Figures

Supplementary Figure 1

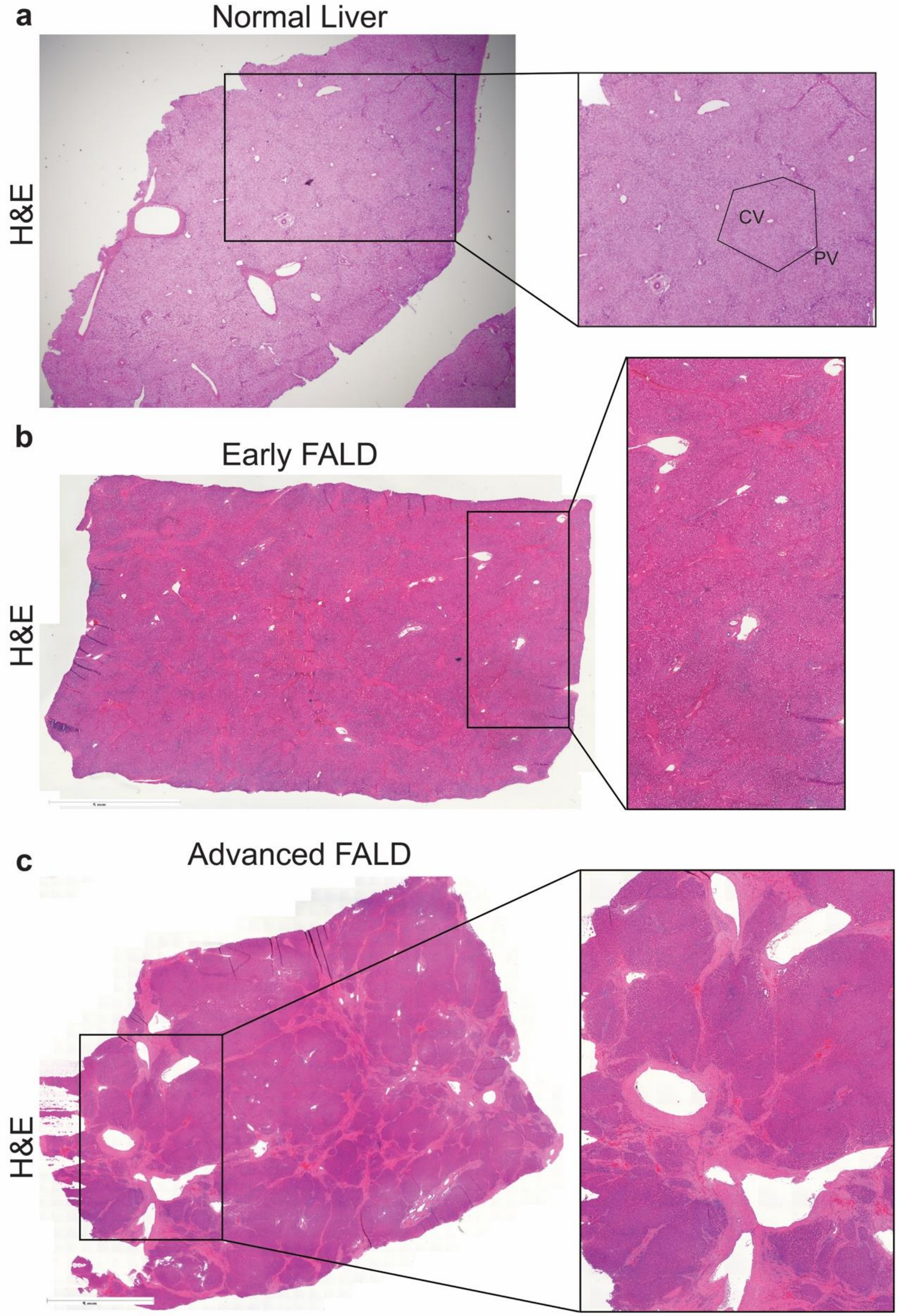

Supplementary Figure 1: H&E staining of normal liver, early, and advanced FALD tissue sections corresponding to regions analyzed by CosMx™ Spatial Transcriptomics Platform (related to Figure 1).

(a) **Normal Liver:** The normal liver sample used for spatial transcriptomics analysis shows the presence of a well-defined hepatic lobule (inset) with a portal vein (PV) and a central vein (CV).
(b) **Early FALD:** Liver explant from a 59-year-old FALD patient undergoing heart-liver transplant showing dilated central veins and areas of bridging fibrosis. Serum liver function tests were normal.
(c) **Advanced FALD:** Liver explant from a 21-year-old patient with extensive liver fibrosis undergoing heart-liver transplantation shows thick bands of bridging fibrosis. Clinically, the patient presented with ascites and abnormal synthetic liver function tests, including decreased albumin and elevated coagulation profile.

Supplementary Figure 2

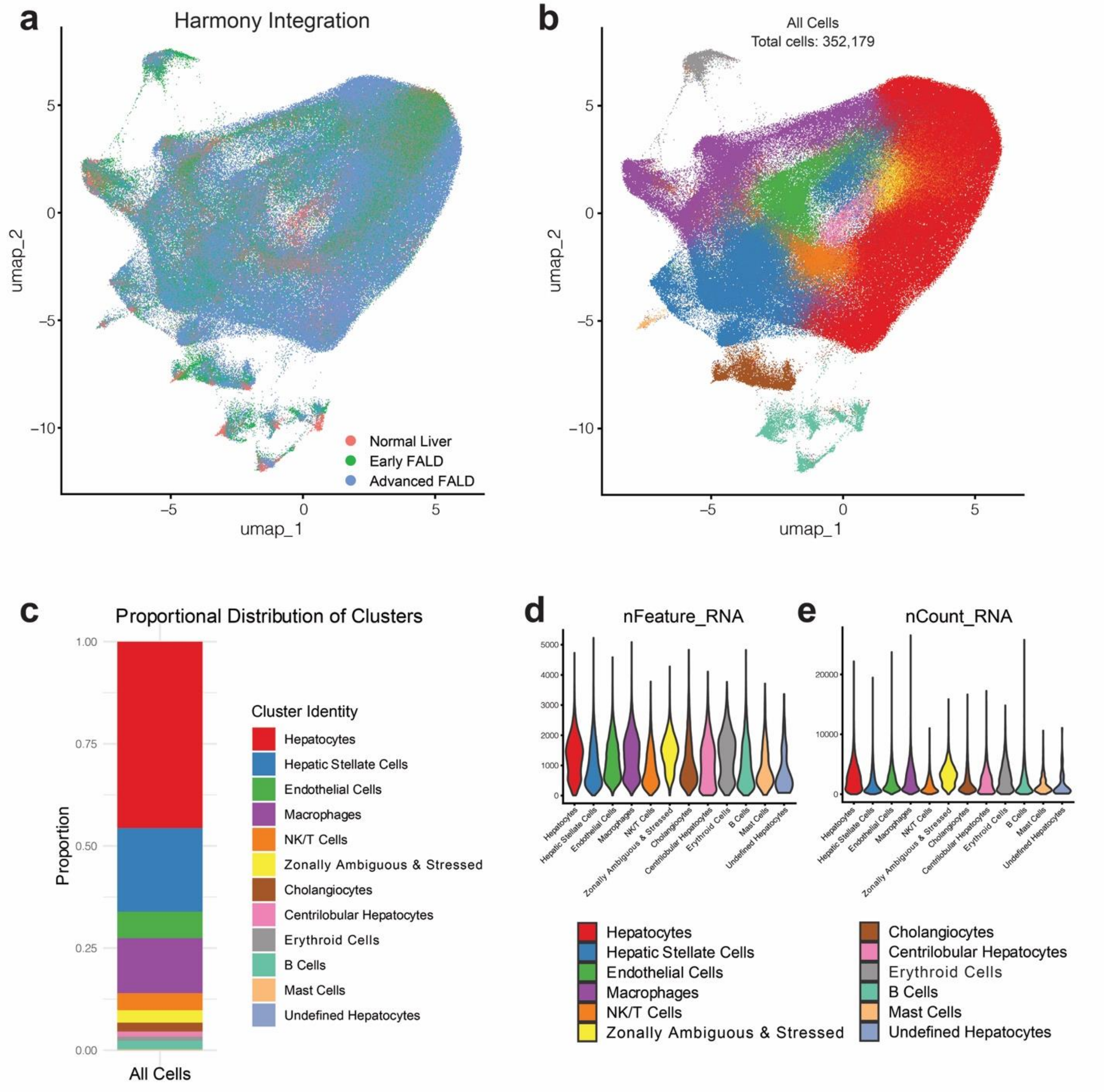

Supplementary Figure 2. Data integration and quality control metrics from CosMx™ Spatial Molecular Imager (SMI) platform from Fontan-associated liver disease (FALD) tissue sections (related to Figure 1).

(a) Uniform manifold approximation and projection (UMAP) following harmony integration pipeline with cells annotated by pathology: normal liver and FALD with early and advanced fibrosis.
(b) UMAP with cells colored by cell type annotation in (c).
(c) Stacked bar plot indicating relative cell proportions across the whole dataset colored by cell type annotation.
(d) Violin plot of nFeature_RNA annotated by cell type, indicating the number of unique genes in each cell.
(e) Violin plot of nCount_RNA annotated by cell type, indicating the number of unique molecular identifiers in each cell.

Supplementary Figure 3

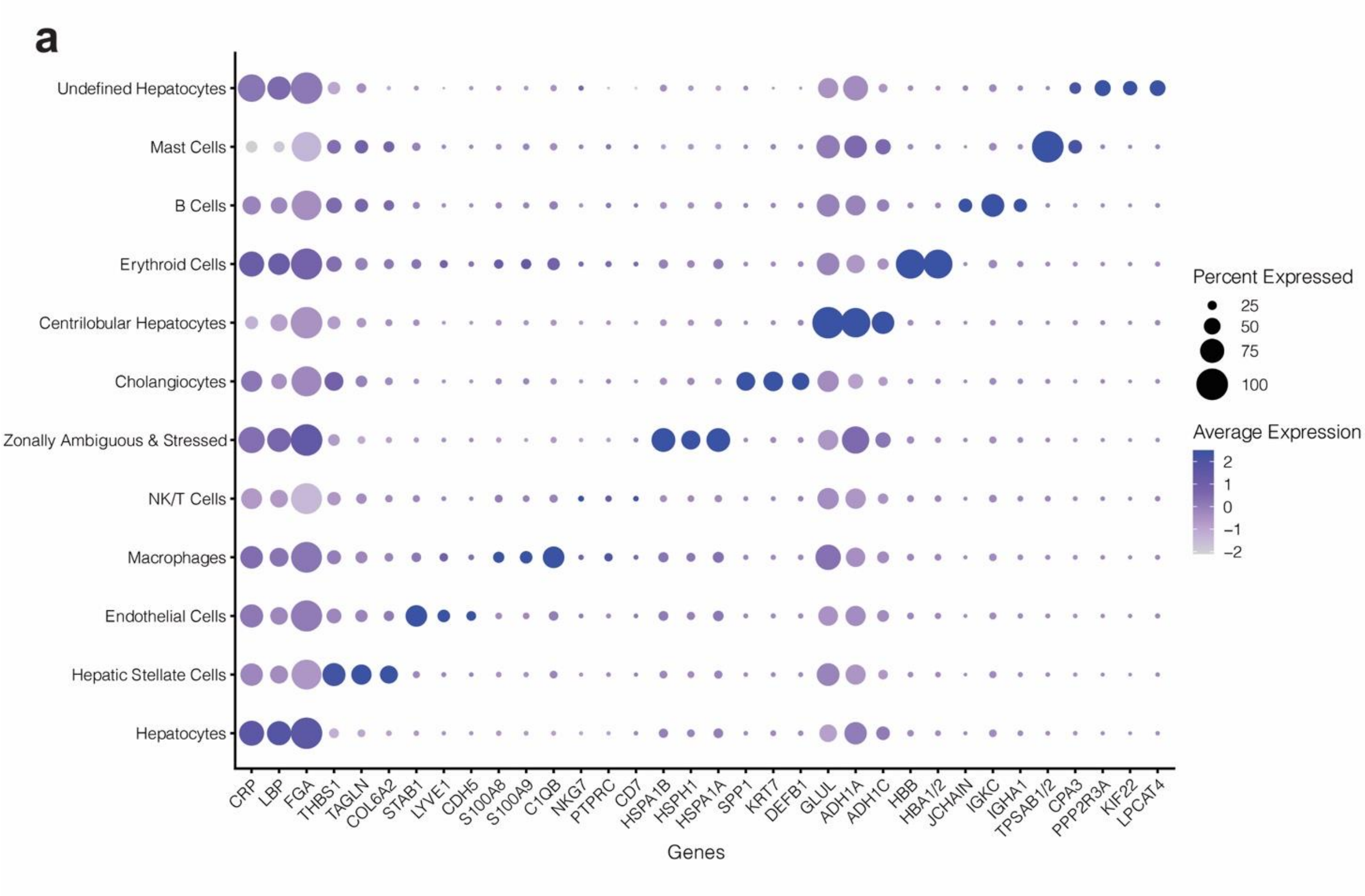

b

	Normal Liver (n)	Early FALD (n)	Advanced FALD (n)	Normal Liver (%)	Early FALD (%)	Advanced FALD (%)
**Hepatocytes**	29036	41785	97289	48.12	48.70	47.22
**Hepatic Stellate Cells**	8936	16830	46300	14.81	19.62	22.47
**Endothelial Cells**	2031	3339	10085	3.37	3.89	4.89
**Macrophages**	9254	13480	24613	15.34	15.71	11.95
**NK/T Cells**	3540	3211	7947	5.87	3.74	3.86
**Zonally Ambiguous & Stressed Hepatocytes**	437	448	9883	0.72	0.52	4.80
**Cholangiocytes**	1377	2664	3629	2.28	3.11	1.76
**Central Vein Hepatocytes**	2562	327	1319	4.25	0.38	0.64
**Erythroid Cells**	506	1668	1496	0.84	1.94	0.73
**B Cells**	2253	1921	3173	3.73	2.24	1.54
**Mast Cells**	398	112	228	0.66	0.13	0.11
**Undefined Hepatocytes**	9	10	83	0.01	0.01	0.04
Total	60339	85795	206045	100	100	100

Supplementary Figure 3: Cell type cluster analysis of sc-transcriptomics samples (related to Figure 1).

(a) Dotplot illustrating expression of various marker genes used to annotate the cell populations.
(b) Absolute number of cells and their corresponding percentages for normal liver, early FALD, and advanced FALD samples.

Supplementary Figure 4

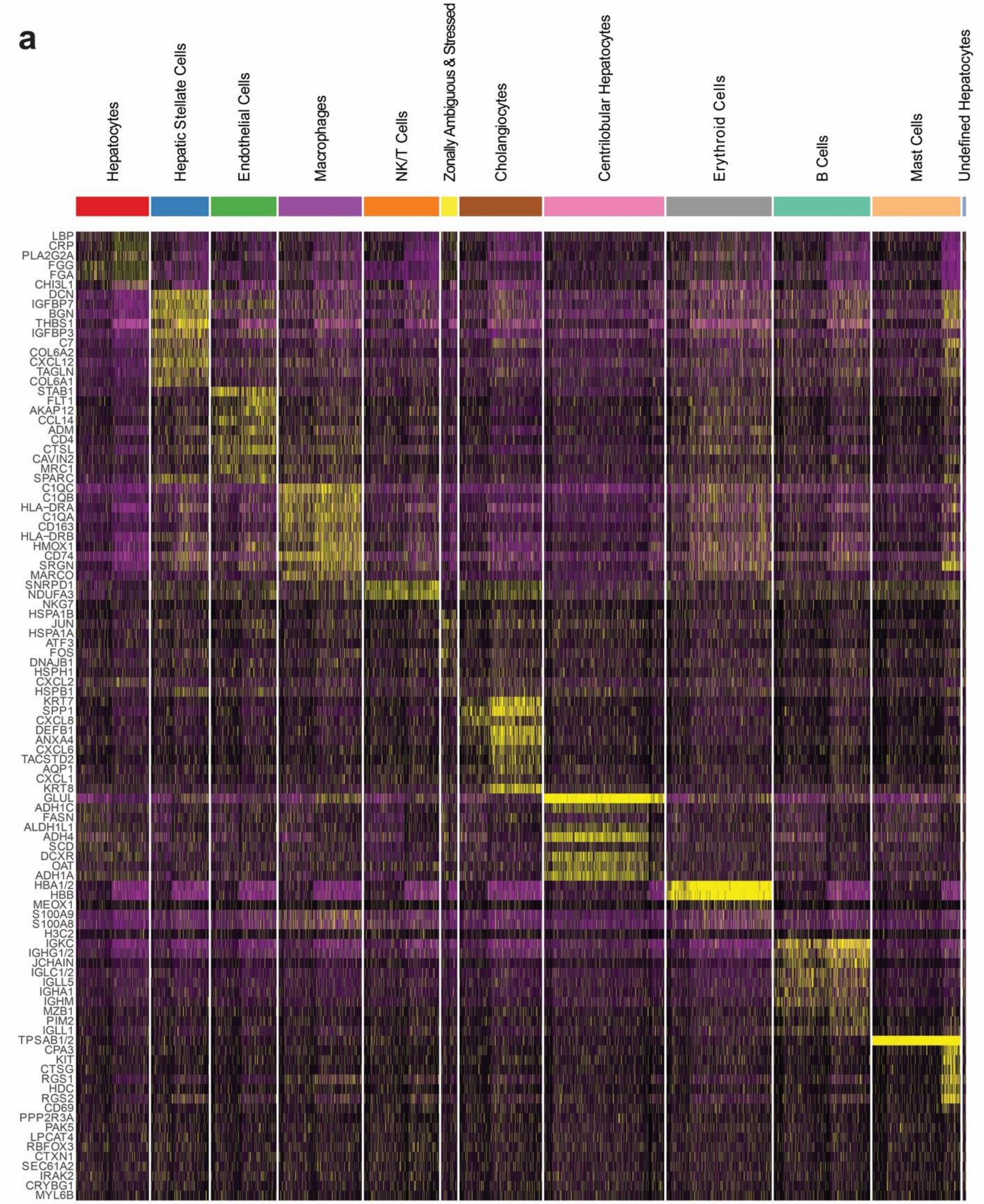

Supplementary Figure 4. Heatmap of cell type marker genes (related to Figure 1).

(a) Heatmap demonstrating relative expression of various marker genes per annotated cell type.

Supplementary Figure 5

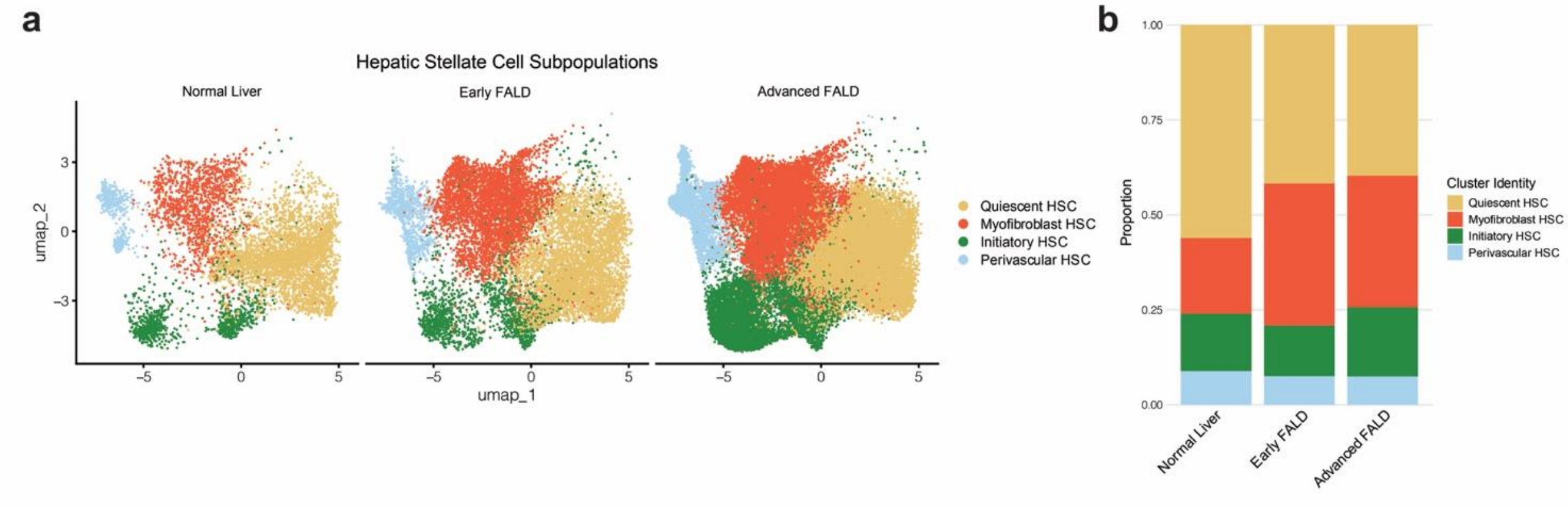

c

gene	avg_log2FC	pct.1	pct.2	cluster
**IGFBP3**	2.289324213	0.947	0.539	Quiescent HSC
**MASP1**	1.918657678	0.414	0.127	Quiescent HSC
**HGF**	1.445505501	0.443	0.193	Quiescent HSC
**RELN**	2.196368806	0.333	0.088	Quiescent HSC
**CXCL12**	1.606910837	0.916	0.677	Quiescent HSC
**MARCKS**	0.822774841	0.517	0.314	Quiescent HSC
**CTSL**	1.209515355	0.427	0.234	Quiescent HSC
**MEG3**	1.29907268	0.353	0.163	Quiescent HSC
**COL6A1**	0.732261162	0.698	0.508	Quiescent HSC
**C7**	2.40055596	0.95	0.505	Myofibroblast HSC
**FBLN5**	1.397964138	0.477	0.237	Myofibroblast HSC
**MFAP4**	1.13772878	0.504	0.279	Myofibroblast HSC
**RGS2**	1.259204959	0.469	0.262	Myofibroblast HSC
**LUM**	1.355692499	0.443	0.241	Myofibroblast HSC
**LAMC3**	1.868341736	0.258	0.085	Myofibroblast HSC
**FBLN1**	1.285785825	0.334	0.167	Myofibroblast HSC
**PDGFRA**	1.054115916	0.389	0.225	Myofibroblast HSC
**GAS1**	1.485934962	0.311	0.148	Myofibroblast HSC
**MGP**	1.153414994	0.328	0.175	Myofibroblast HSC
**C7**	2.40055596	0.95	0.505	Myofibroblast HSC
**FBLN5**	1.397964138	0.477	0.237	Myofibroblast HSC
**MYH11**	2.445442646	0.378	0.153	Initiatory HSC
**TAGLN**	1.578647025	0.82	0.653	Initiatory HSC
**THBS1**	0.620346713	0.897	0.735	Initiatory HSC
**TPM2**	1.73004641	0.395	0.245	Initiatory HSC
**ACTA2**	1.826912304	0.416	0.266	Initiatory HSC
**MYL9**	1.493922764	0.805	0.666	Initiatory HSC
**DES**	2.600436547	0.197	0.059	Initiatory HSC
**ACTG2**	2.203640267	0.18	0.065	Initiatory HSC
**FLNA**	1.462186706	0.427	0.338	Initiatory HSC
**OGN**	1.777070131	0.152	0.067	Initiatory HSC
**RGS5**	4.477786514	0.714	0.094	Perivascular HSC
**PDK4**	2.657180337	0.656	0.283	Perivascular HSC
**SPARCL1**	2.425259835	0.54	0.175	Perivascular HSC
**APOLD1**	2.407801047	0.515	0.182	Perivascular HSC
**ATP1B3**	2.131984942	0.592	0.291	Perivascular HSC
**TM4SF1**	2.287640326	0.468	0.19	Perivascular HSC
**CLDN5**	3.013872574	0.332	0.077	Perivascular HSC
**BCAM**	2.278099783	0.348	0.095	Perivascular HSC
**ADAMTS9**	1.864862625	0.431	0.182	Perivascular HSC
**ADIRF**	1.872790494	0.388	0.151	Perivascular HSC

Supplementary Figure 5: Subtypes of hepatic stellate cells (related to Figure 1).

(a) UMAP of hepatic stellate cells (HSC) distribution across normal liver, early FALD, and advanced FALD.
(b) Bar graph distribution of HSC subtypes by disease pathology.
(c) Top 10 markers for each of the HSC subtypes.

Supplementary Figure 6

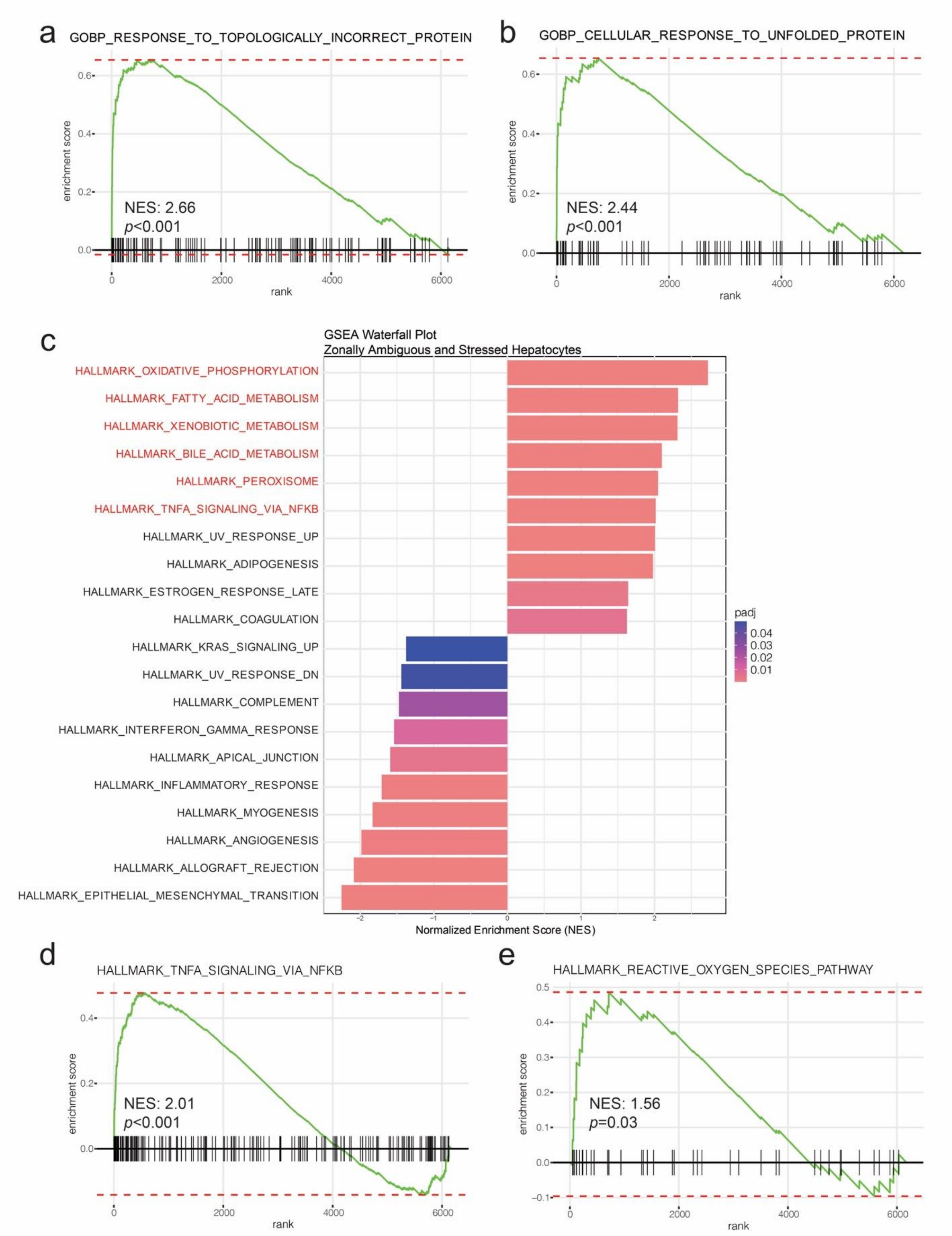

Supplementary Figure 6. Zonally ambiguous and stressed hepatocytes demonstrate upregulation of pathways related to unfolded protein response and cellular stress (related to Figure 2).

(a) Gene set enrichment analysis (GSEA) running enrichment score plot for gene ontology pathway: response to topologically incorrect protein.
(b) GSEA running enrichment score plot for gene ontology pathway: cellular response to unfolded protein.
(c) Waterfall plot of top 10 upregulated and downregulated hallmark pathways by normalized enrichment score (NES) and adjusted p-value.
(d) GSEA running enrichment score plot for hallmark pathway: TNFα signaling via NKκB.
(e) GSEA running enrichment score plot for hallmark pathway: reactive oxygen species.

Supplementary Figure 7

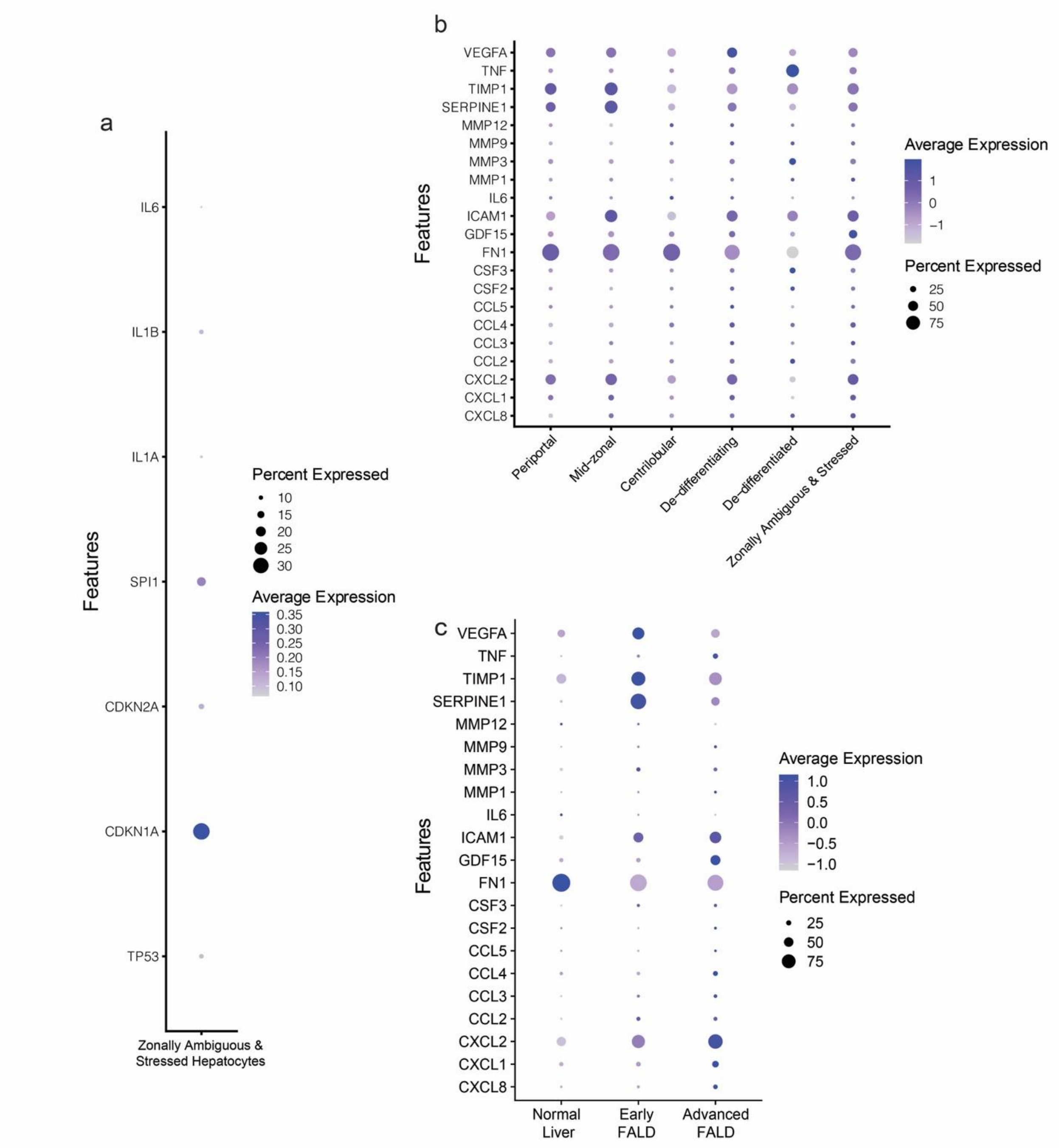

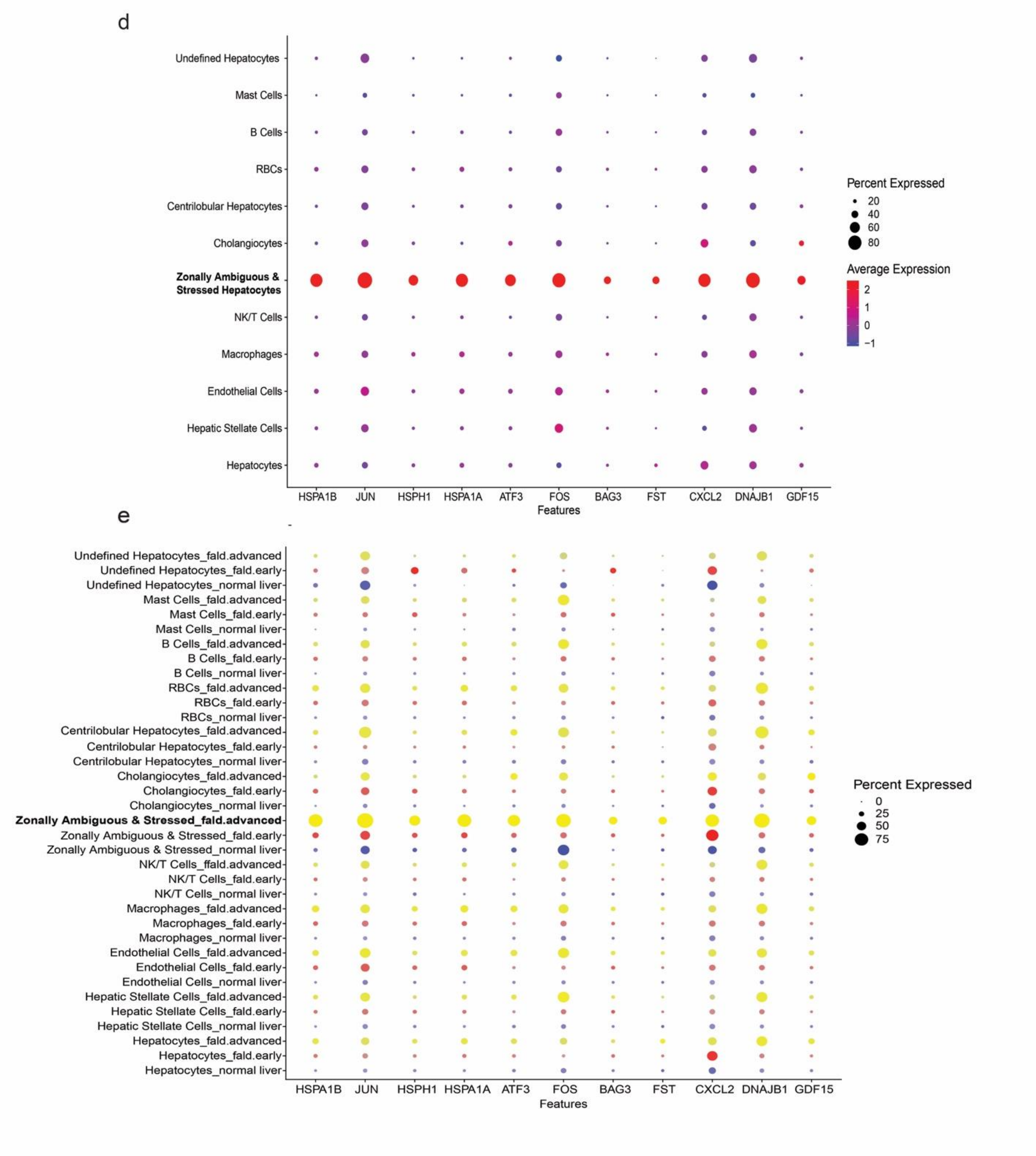
d
Undefined Hepatocytes
Mast Cells
B Cells
RBCs
Centrilobular Hepatocytes
Cholangiocytes
Zonally Ambiguous & Stressed Hepatocytes
NK/T Cells
Macrophages
Endothelial Cells
Hepatic Stellate Cells
Hepatocytes
HSPA1B
JUN
HSPH1
HSPA1A
ATF3
FOS
BAG3
FST
CXCL2
DNAJB1
GDF15
Features
Percent Expressed
20
40
60
80
Average Expression
2
1
0
−1
e
Undefined Hepatocytes_fald.advanced
Undefined Hepatocytes_fald.early
Undefined Hepatocytes_normal liver
Mast Cells_fald.advanced
Mast Cells_fald.early
Mast Cells_normal liver
B Cells_fald.advanced
B Cells_fald.early
B Cells_normal liver
RBCs_fald.advanced
RBCs_fald.early
RBCs_normal liver
Centrilobular Hepatocytes_fald.advanced
Centrilobular Hepatocytes_fald.early
Centrilobular Hepatocytes_normal liver
Cholangiocytes_fald.advanced
Cholangiocytes_fald.early
Cholangiocytes_normal liver
Zonally Ambiguous & Stressed_fald.advanced
Zonally Ambiguous & Stressed_fald.early
Zonally Ambiguous & Stressed_normal liver
NK/T Cells_ffald.advanced
NK/T Cells_fald.early
NK/T Cells_normal liver
Macrophages_fald.advanced
Macrophages_fald.early
Macrophages_normal liver
Endothelial Cells_fald.advanced
Endothelial Cells_fald.early
Endothelial Cells_normal liver
Hepatic Stellate Cells_fald.advanced
Hepatic Stellate Cells_fald.early
Hepatic Stellate Cells_normal liver
Hepatocytes_fald.advanced
Hepatocytes_fald.early
Hepatocytes_normal liver
HSPA1B
JUN
HSPH1
HSPA1A
ATF3
FOS
BAG3
FST
CXCL2
DNAJB1
GDF15
Features
Percent Expressed
0
25
50
75

Supplementary Figure 7. Zonally ambiguous and stressed hepatocytes upregulate SASP markers in FALD (related to Figure 2).

(a) Dotplot illustrating expression of various senescence marker genes.
(b) Dotplot illustrating the expression of SASP markers across hepatocyte subtypes (hepatocyte subtypes defined in Figure 3b).
(c) Dotplot analysis shows that expression of SASP markers increase with early and advanced FALD as compared to normal liver.
(d) Dotplot analysis shows that zonally ambiguous and stressed hepatocyte module genes are uniquely upregulated in this cell type.
(e) Dotplot analysis illustrating that expression of zonally ambiguous and stressed hepatocyte module genes is uniquely upregulated in advanced FALD.

Supplementary Figure 8

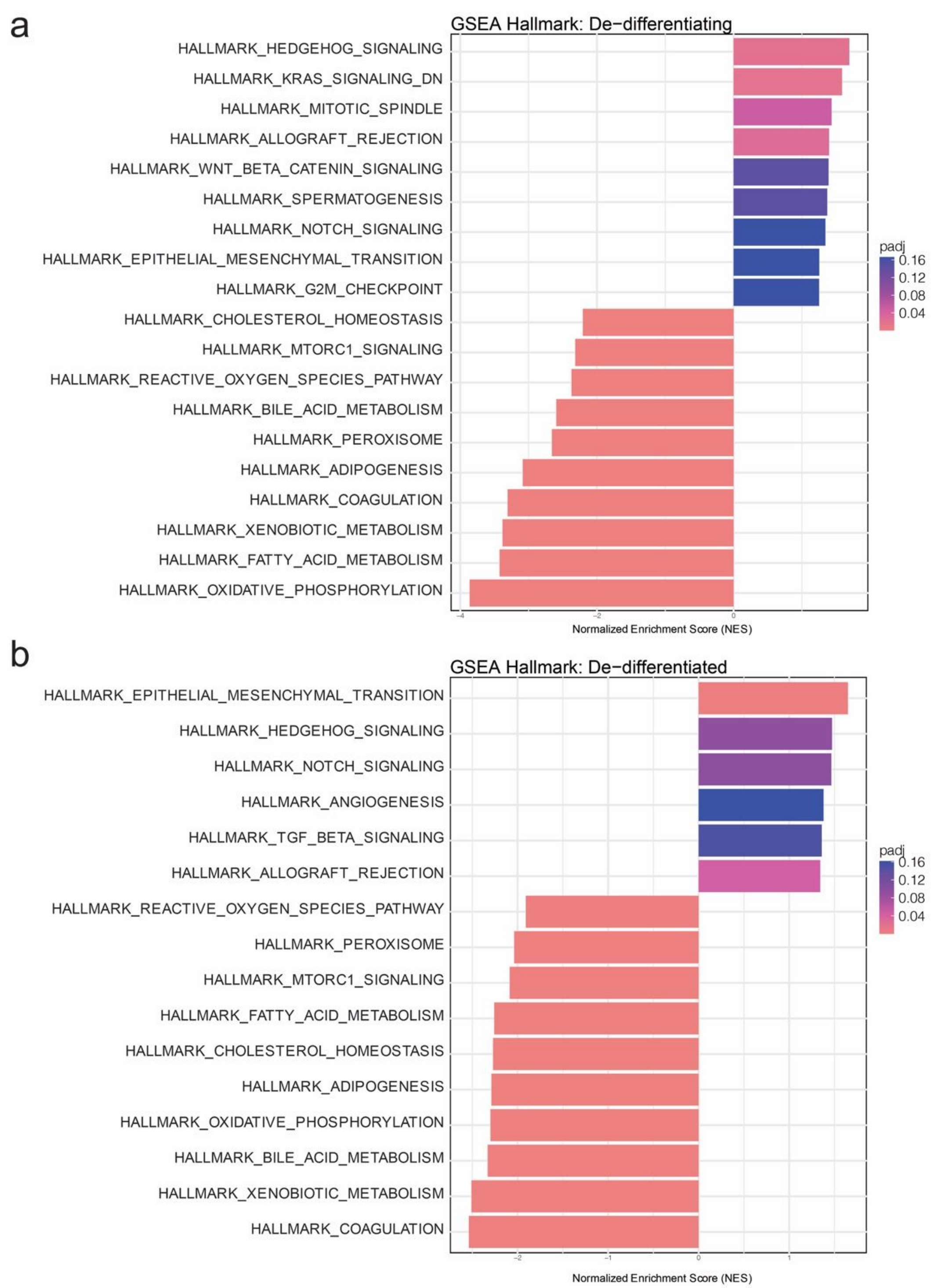

Supplementary Figure 8. GSEA of De-differentiating and De-differentiated hepatocytes following sub-clustering (related to Figure 3).

(a) Waterfall plot of top 10 upregulated and downregulated hallmark pathways by normalized enrichment score (NES) and adjusted p-value for de-differentiating hepatocytes.

(b) Waterfall plot of top 10 upregulated and downregulated hallmark pathways by normalized enrichment score (NES) and adjusted p-value for de-differentiated hepatocytes.

Supplementary Figure 9

a

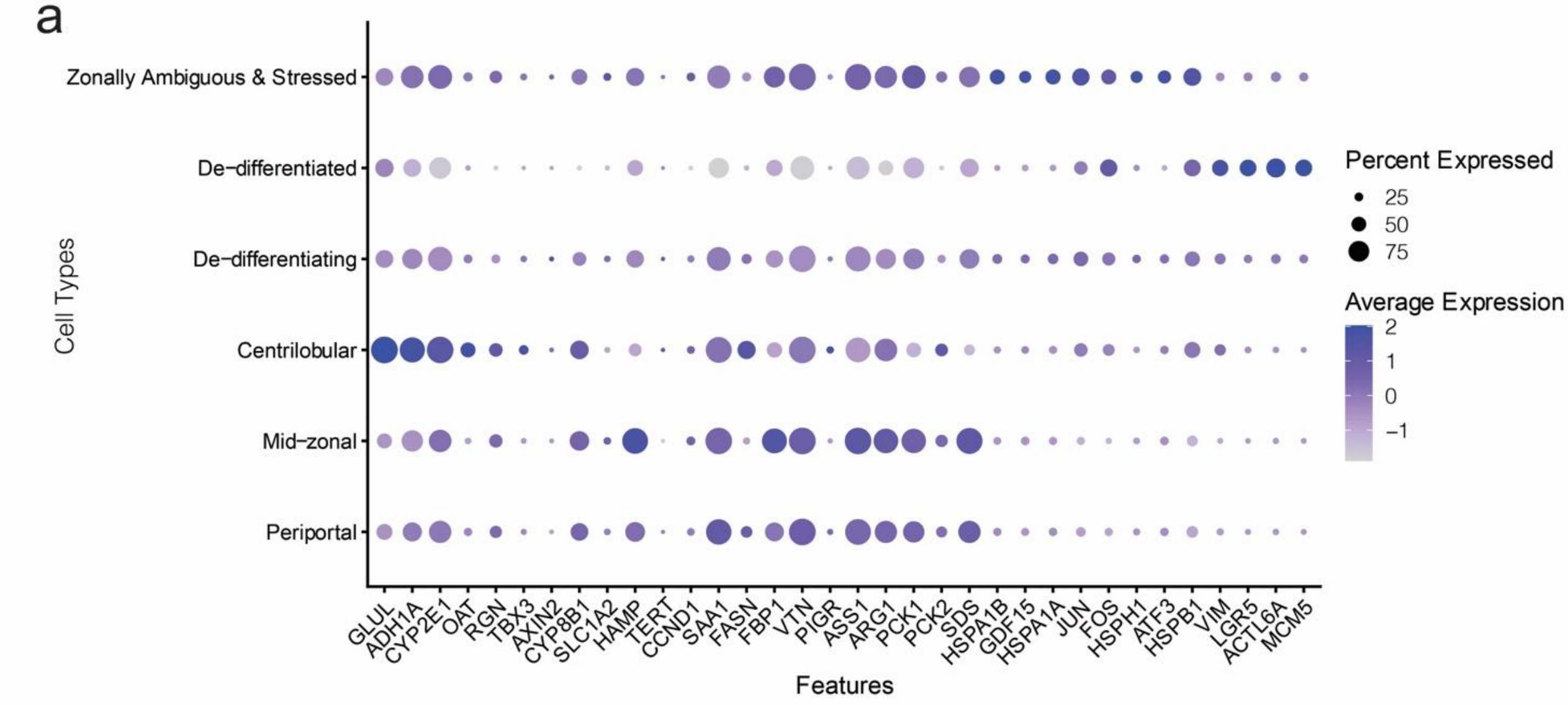

Supplementary Figure 9. Cell-type markers for hepatocyte subpopulations (related to Figure 3).

(a) Dotplot illustrating expression of various marker genes used to annotate the hepatocyte subpopulations.

Supplementary Figure 10

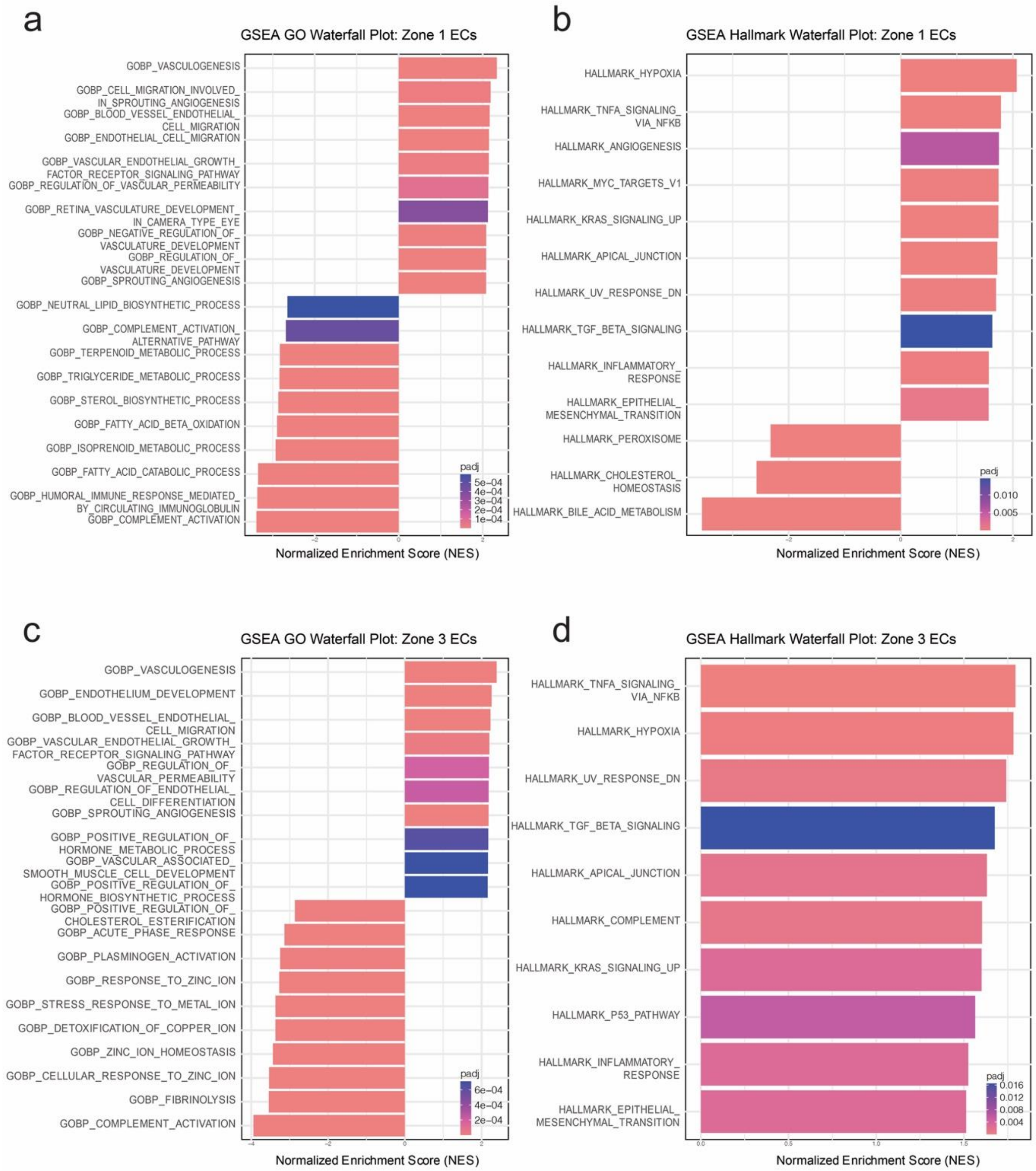

Supplementary Figure 10. Gene set enrichment analysis of zone 1 and zone 3 endothelial cells (related to Figure 4).

(a) Waterfall plot of the top 10 upregulated and downregulated gene ontology pathways by normalized enrichment score (NES) and adjusted p-value for zone 1 endothelial cells.
(b) Waterfall plot of the top 10 upregulated and downregulated hallmark pathways by normalized enrichment score (NES) and adjusted p-value for zone 1 endothelial cells.
(c) Waterfall plot of the top 10 upregulated and downregulated gene ontology pathways by normalized enrichment score (NES) and adjusted p-value for zone 3 endothelial cells.
(d) Waterfall plot of the top 10 upregulated and downregulated hallmark pathways by normalized enrichment score (NES) and adjusted p-value for zone 3 endothelial cells.

Supplementary Figure 11

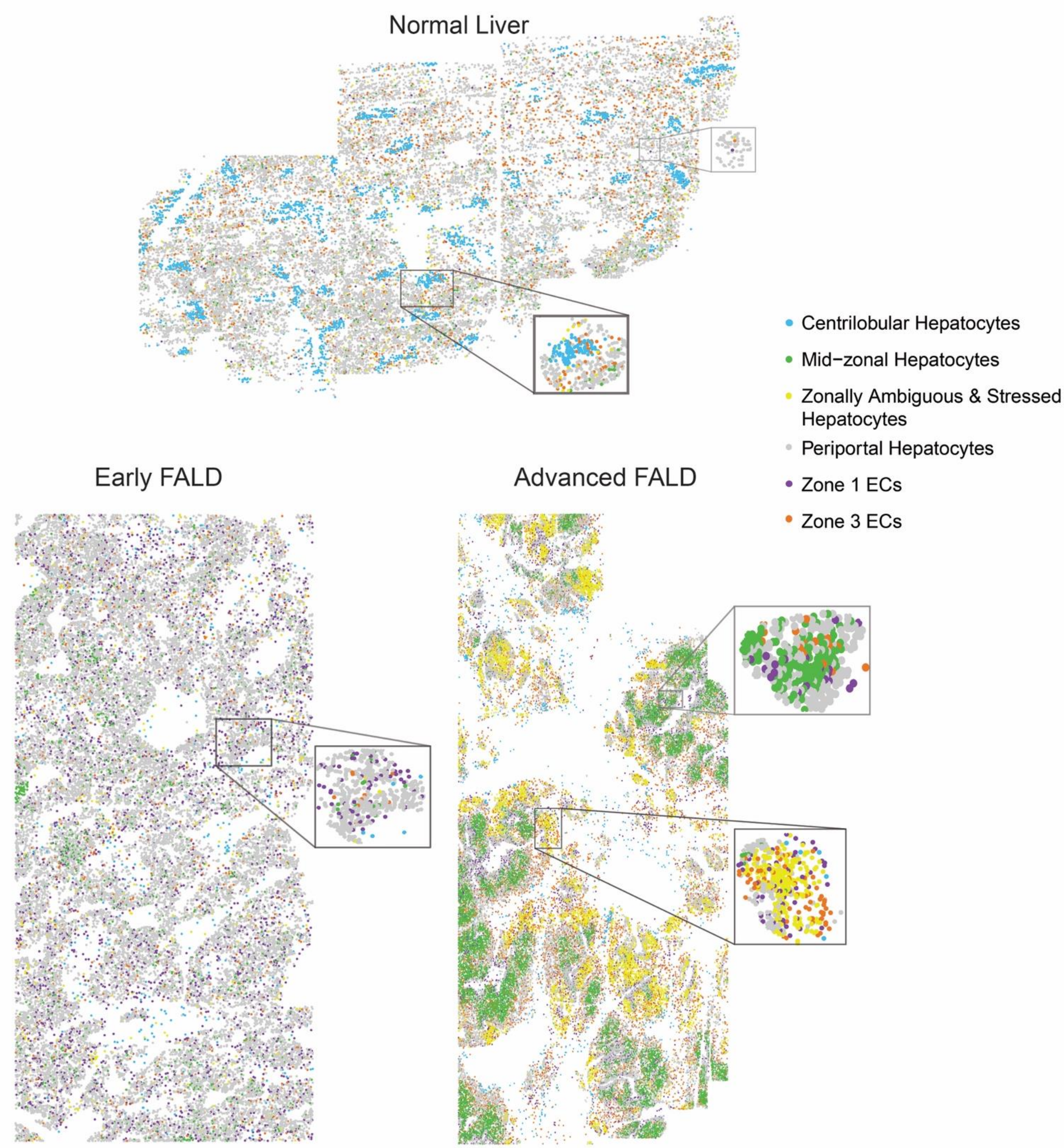

Supplementary Figure 11. Spatial feature plots of zone 1 and zone 3 endothelial cells (related to Figure 4).

(a) Spatial plots of zone 1 and 3 endothelial cells (ECs) and their spatial correlation with hepatocyte subpopulations in normal liver, early FALD, and advanced FALD liver samples.

Supplementary Figure 12

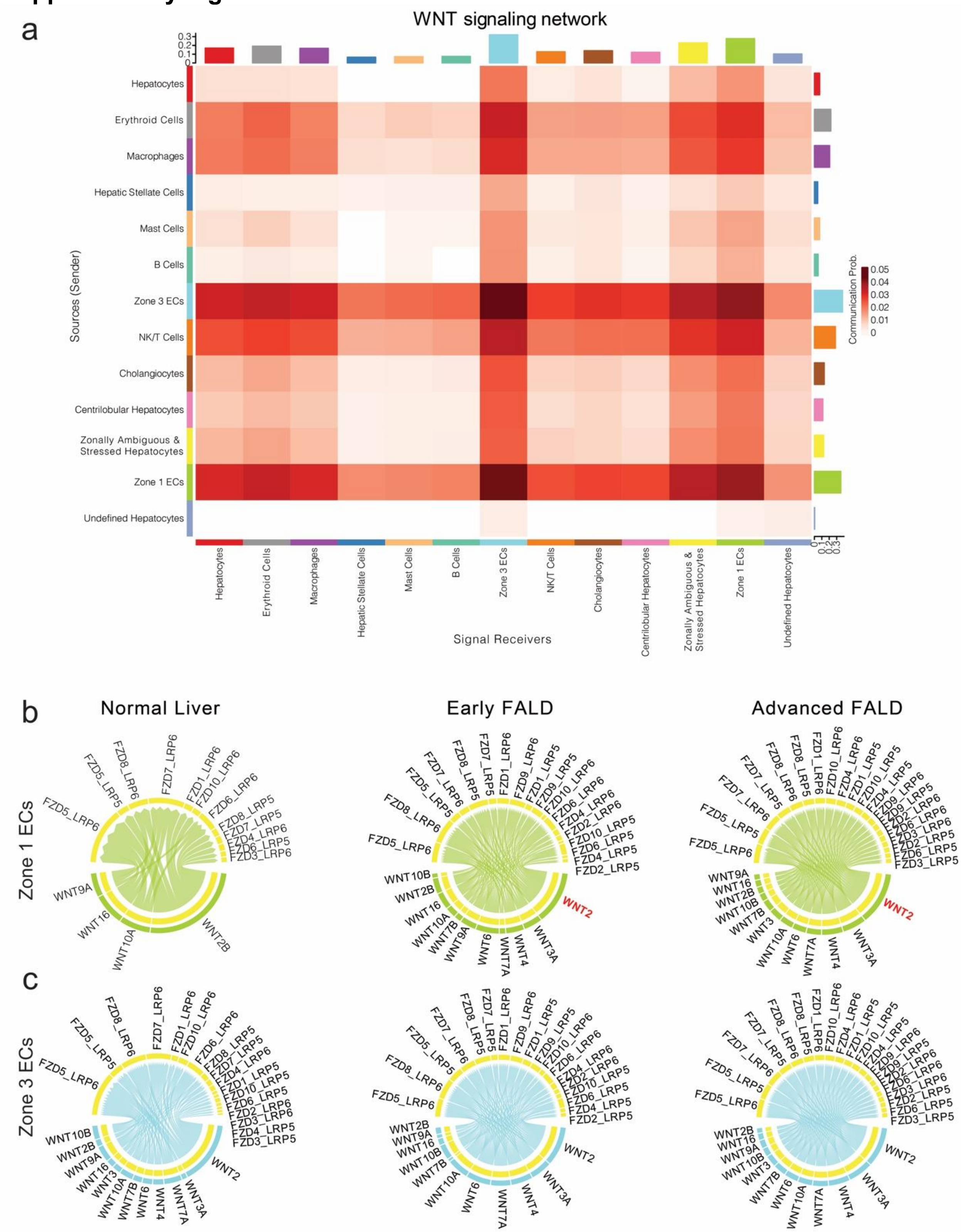

Supplementary Figure 12. CellChat analysis demonstrates increased probability of Wnt signals from endothelial cells to zonally ambiguous and stressed hepatocytes (related to Figure 5).

(a) Heatmap of cellular communication for WNT signaling across the entire dataset.
(b) Chord diagram of WNT-FZD signaling between zone 1 endothelial cells to zonally ambiguous and stressed hepatocytes across disease pathology.
(c) Chord diagram of WNT-FZD signaling between zone 3 endothelial cells to zonally ambiguous and stressed hepatocytes across disease pathology.

Supplementary Figure 13

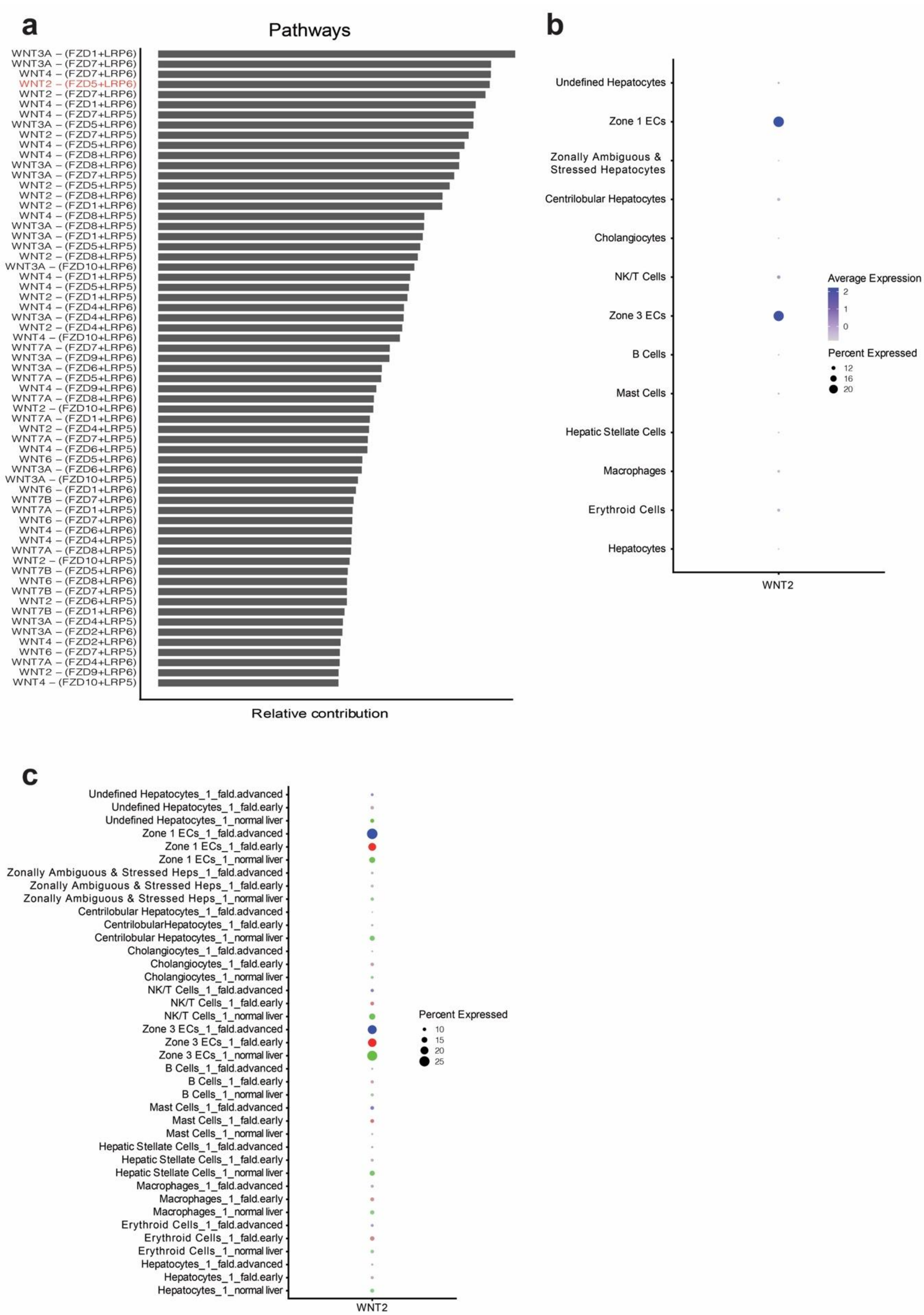

Supplementary Figure 13. WNT2 shows increased expression in zone 1 endothelial cells in advanced FALD (related to Figure 5).

(a) Waterfall plot showing relative contribution of all WNT-FZD pairs in entire dataset.
(b) Dotplot of WNT2 across all cell types in CosMx^{TM} dataset.
(c) Dotplot of WNT2 across all cell types and disease pathology in CosMx^{TM} dataset.

Supplementary Figure 14

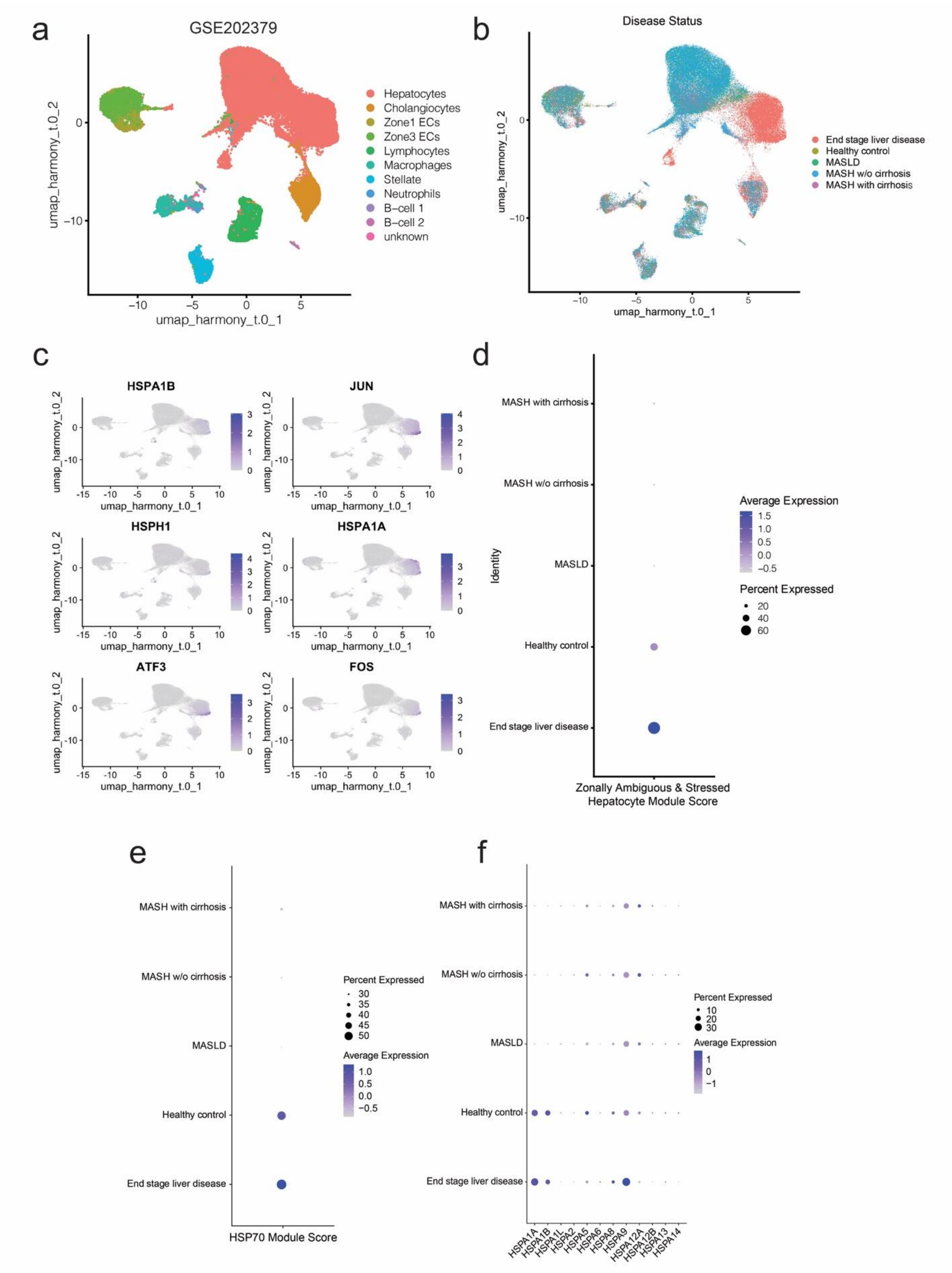

Supplementary Figure 14. Zonally ambiguous and stressed hepatocyte markers are identified in hepatocytes in end-stage liver disease (related to Figure 5).

(a) Uniform manifold approximation and projection (UMAP) of cell types from GSE202379. We subclustered the endothelial cells in this dataset for zone 1 and zone 3 endothelial cells.
(b) UMAP of disease pathology from GSE202379.
(c) Feature plot of expression of top marker genes from zonally ambiguous and stressed hepatocytes.
(d) Dotplot of gene expression module score for zonally ambiguous and stressed hepatocytes based on top 10 differentially expressed genes. Genes for the module score are the following: *HSPA1B, JUN, HSPH1, HSPA1A, ATF3, FOS, BAG3, FST, CXCL2, DNAJB1,* and *GDF15*.
(e) Dotplot of gene expression module score for HSP70 using marker genes in (f).
(f) Dotplot of gene expression of individual HSP70 subunit genes comprising the genes in the HSP70 module score.

Supplementary Figure 15

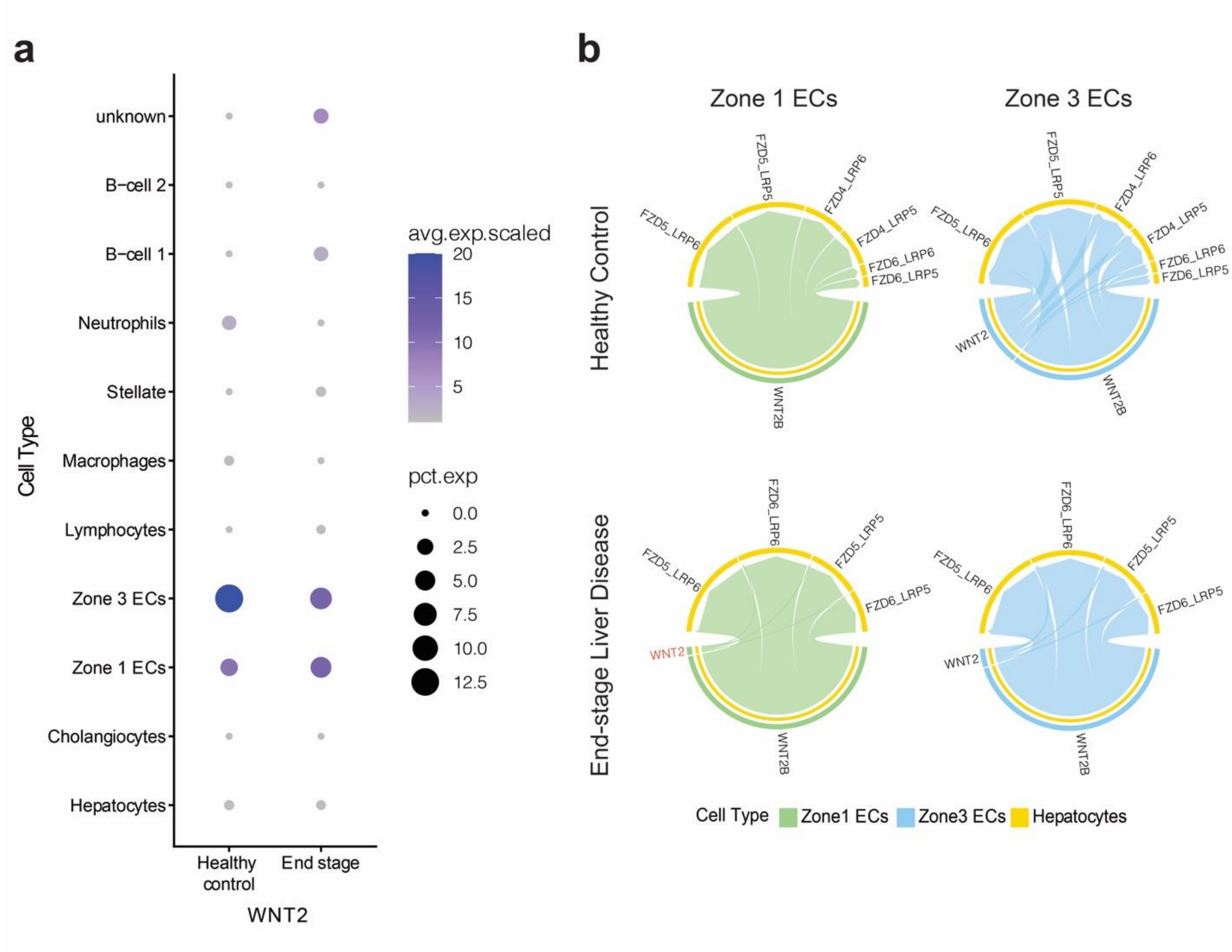

Supplementary Figure 15. Ectopic WNT2 signaling from zone 1 endothelial cells to hepatocytes in end-stage liver disease (related to Figure 5).

(a) Dotplot of WNT2 gene expression changes in various cell populations in healthy control and end-stage liver disease samples from GSE202379.

(b) Chord diagram of WNT-FZD signaling between zone 1 endothelial cells and hepatocytes and zone 3 endothelial cells and hepatocytes for both healthy control and end-stage liver disease from GSE202379.

Supplementary Figure 16

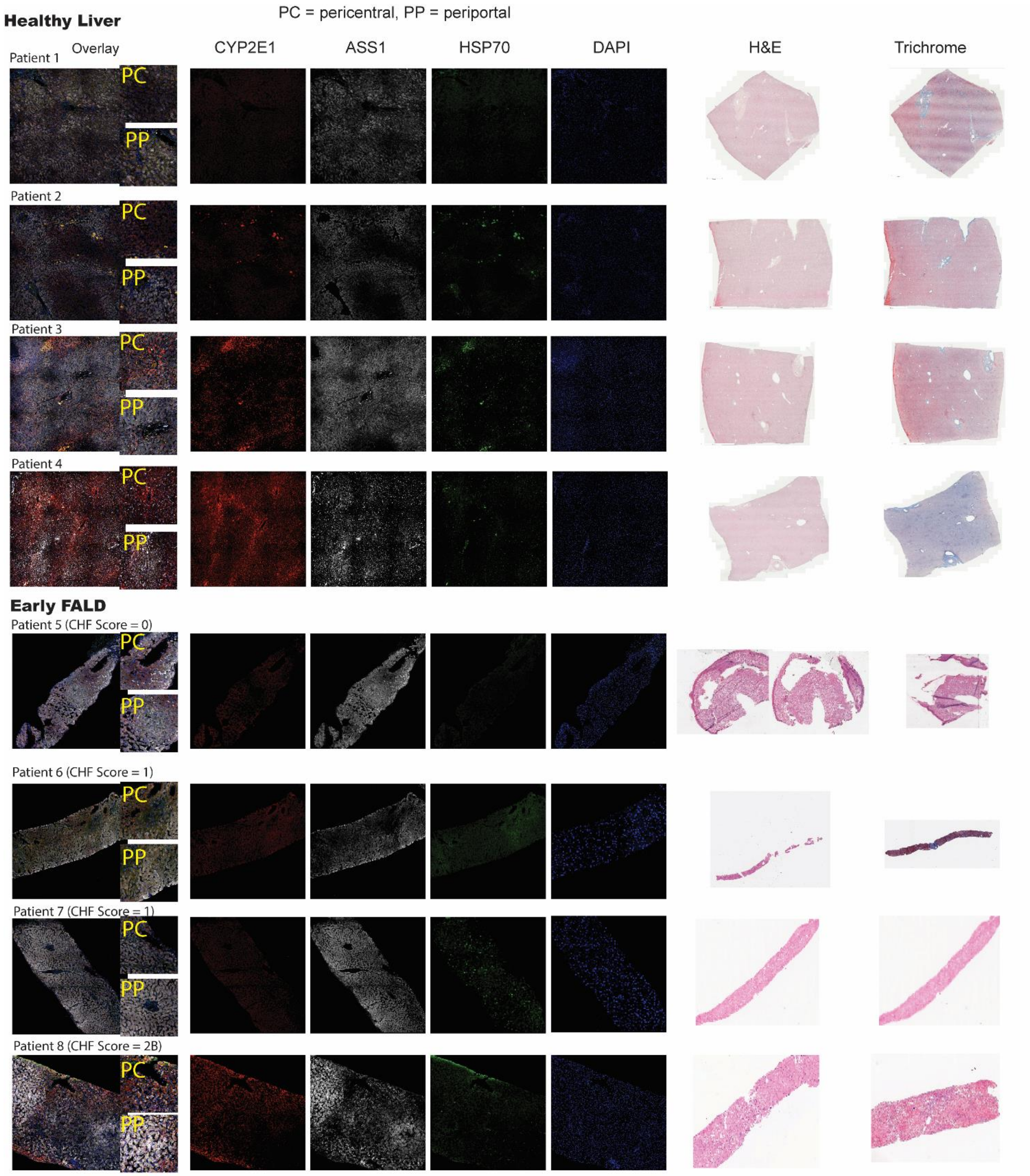

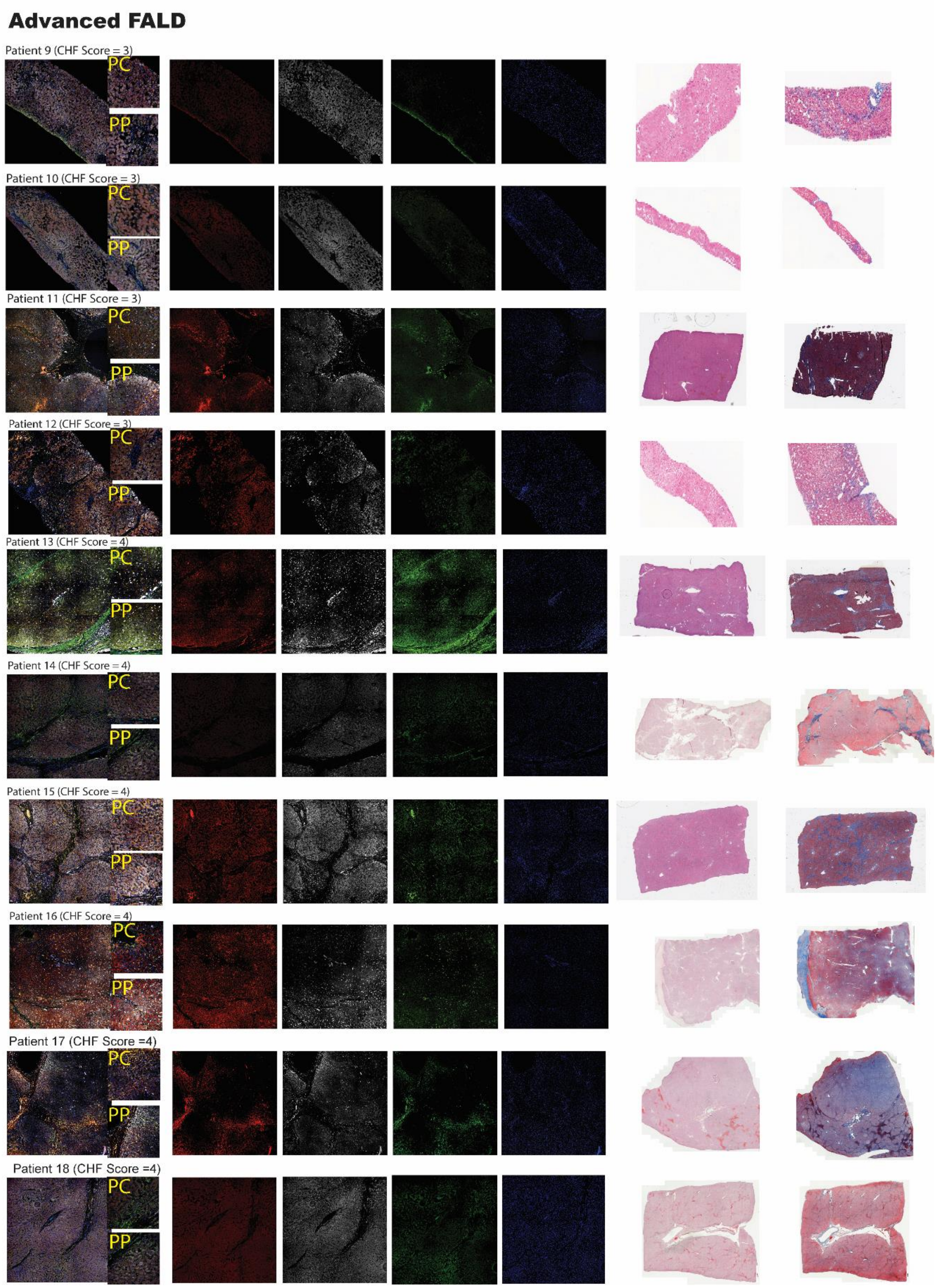
Advanced FALD
Patient 9 (CHF Score = 3)
PC
PP
Patient 10 (CHF Score = 3)
PC
PP
Patient 11 (CHF Score = 3)
PC
PP
Patient 12 (CHF Score = 3)
PC
PP
Patient 13 (CHF Score = 4)
PC
PP
Patient 14 (CHF Score = 4)
PC
PP
Patient 15 (CHF Score = 4)
PC
PP
Patient 16 (CHF Score = 4)
PC
PP
Patient 17 (CHF Score =4)
PC
PP
Patient 18 (CHF Score =4)
PC
PP

Supplementary Figure 16. Immunofluorescence of hepatocyte zonation markers and HSP70 display aberrant zonation and increased HSP70 expression in Early and Advanced FALD. Corresponding H&E and Mason's Trichrome stain to demonstrate fibrosis in FALD liver biopsies and explants.

Patient CHF score provided with each sample. CHF scores of 0-2 were characterized as Early FALD and CHF scores of 3-4 were representative of Advanced FALD.
